# Spatiotemporally deciphering the mysterious mechanism of persistent HPV‐induced malignant transition and immune remodelling from HPV‐infected normal cervix, precancer to cervical cancer: Integrating single‐cell RNA‐sequencing and spatial transcriptome

**DOI:** 10.1002/ctm2.1219

**Published:** 2023-03-26

**Authors:** Chenyan Guo, Xinyu Qu, Xiaoyan Tang, Yu Song, Jue Wang, Keqin Hua, Junjun Qiu

**Affiliations:** ^1^ Department of Gynecology Obstetrics and Gynecology Hospital Fudan University Shanghai China; ^2^ Shanghai Key Laboratory of Female Reproductive Endocrine‐Related Diseases Shanghai China

**Keywords:** cervical cancer, single cell, spatial transcriptome

## Abstract

**Background:**

The mechanism underlying cervical carcinogenesis that is mediated by persistent human papillomavirus (HPV) infection remains elusive.

**Aims:**

Here, for the first time, we deciphered both the temporal transition and spatial distribution of cellular subsets during disease progression from normal cervix tissues to precursor lesions to cervical cancer.

**Materials & Methods:**

We generated scRNA‐seq profiles and spatial transcriptomics data from nine patient samples, including two HPV‐negative normal, two HPV‐positive normal, two HPV‐positive HSIL and three HPV‐positive cancer samples.

**Results:**

We not only identified three ‘HPV‐related epithelial clusters’ that are unique to normal, high‐grade squamous intraepithelial lesions (HSIL) and cervical cancer tissues but also discovered node genes that potentially regulate disease progression. Moreover, we observed the gradual transition of multiple immune cells that exhibited positive immune responses, followed by dysregulation and exhaustion, and ultimately established an immune‐suppressive microenvironment during the malignant program. In addition, analysis of cellular interactions further verified that a ‘homeostasis‐balance‐malignancy’ change occurred within the cervical microenvironment during disease progression.

**Discussion:**

We for the first time presented a spatiotemporal atlas that systematically described the cellular heterogeneity and spatial map along the four developmental steps of HPV‐related cervical oncogenesis, including normal, HPV‐positive normal, HSIL and cancer. We identified three unique HPV‐related clusters, discovered critical node genes that determined the cell fate and uncovered the immune remodeling during disease escalation.

**Conclusion:**

Together, these findings provided novel possibilities for accurate diagnosis, precise treatment and prognosis evaluation of patients with precancer and cervical cancer.

## Graphical abstract

- Three HPV‐related epithelial clusters unique to HPV infection, HSIL and cervical cancer were identified.
- We discovered several critical node genes that potentially determined disease progression.
- We observed a gradual transition of multiple immune cells from a positive immune response to dysregulation and exhaustion, leading to an immune‐suppressive microenvironment of cervical cancer.

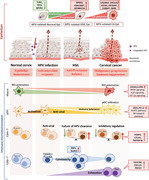

## 1 INTRODUCTION

Cervical cancer (CC) is the fourth most frequently diagnosed cancer and the fourth leading cause of cancer death in women, with a global annual incidence of ∼604,000 cases.^1^ Despite advances in clinical management, up to 30% of patients die from the disease, resulting in a disproportionately high burden worldwide.^2^ It is well known that CC arises via a series of four key steps – human papillomavirus (HPV) transmission, viral persistence, progression of a clone of persistently infected cells to precancer and invasion.^3^ Among these processes, persistent HPV infection, mostly with HPV‐16 and HPV‐18, is the most common cause. However, the exact architecture transformation, cellular events and molecular virology underlying the above transition steps from normal cervical tissue to cervical precursor or cancer remain unexplored. Specifically, it is unknown why some HPV infections persist and progress to cancer, whereas other HPV infections are cleared or precancer tissues return to normal states. How persistent high‐risk HPV infections induce the carcinogenic process from high‐grade squamous intraepithelial lesions (HSIL) to cancer also remains unknown. Thus, there is a clear need for a deeper understanding of persistent HPV infection and HPV‐induced malignancy transition.

Over the last few years, single‐cell RNA‐sequencing (scRNA‐seq) has emerged as a powerful tool for uncovering cellular subpopulations and intercellular crosstalk within the tumour microenvironment (TME). The application of scRNA‐seq enabled an individual‐cell‐level view into various cancers, such as breast and ovarian cancer,^4^, ^5^ at unprecedented molecular resolution. However, in the CC field, scRNA‐seq research remains almost absent. Although a previous study from our laboratory initially evaluated transcriptomics and cell compositions using one sample of CC tissue and paired adjacent normal cervical tissue,^6^ the sample size was relatively small, limiting the power to detect true intratumour heterogeneity. Moreover, the carcinogenesis mechanism of persistent HPV infection was not explored, let alone the HPV‐induced malignancy transition from precancer to cancer. Most importantly, the loss of spatial information limits our understanding of cellular interactions and organization in the TME of CC.

The recent advanced spatial transcriptomics (ST) method overcomes the limitation of scRNA‐seq, allowing the mapping of spatial transcripts at single‐cell resolution. The limited number of published studies on melanoma, breast cancer and prostate cancer has created new prospects in cancer research.^7^, ^8^ However, in CC, the spatial dimensions of entire transcriptomes and the TME remain unexplored. Therefore, given the complexity and incomplete understanding of CC development, we established a molecular approach that enables the simultaneous analysis of spatial gene expression patterns and cellular heterogeneity to spatiotemporally decipher the unknown role of persistent HPV infection in the malignancy transition from precursor lesions to CC.

According to previous studies and preliminary exploration of our laboratory, we speculate that after HPV infects cervical epithelium, during the process from normal to precancerous to cancerous, each stage has its own specific HPV‐infected epithelial clusters and has a clear path of cell fate transformation. In addition, the immune microenvironment changes from immune activation to imbalance to exhaustion, and finally immune suppression. Thereby, to verify the previous assumptions, we for the first time presented a spatiotemporal atlas that systematically described the cellular heterogeneity and spatial map along the four developmental steps of HPV‐related CC oncogenesis, including normal, HPV‐positive normal, HSIL and cancer. We identified three unique HPV‐related clusters, discovered critical node genes that determined the cell fate and uncovered the immune remodelling during disease escalation. Overall, our comprehensive atlas of the cervical ecosystem provides deeper insights into the mechanism of persistent HPV infection‐induced cervical carcinogenesis and promising treatment targets for CC.

## 2 RESULTS

### 2.1 Identifying the single‐cell atlas in the normal cervix, precursor lesion and cervical cancer with persistent HPV infection

**FIGURE 1:**
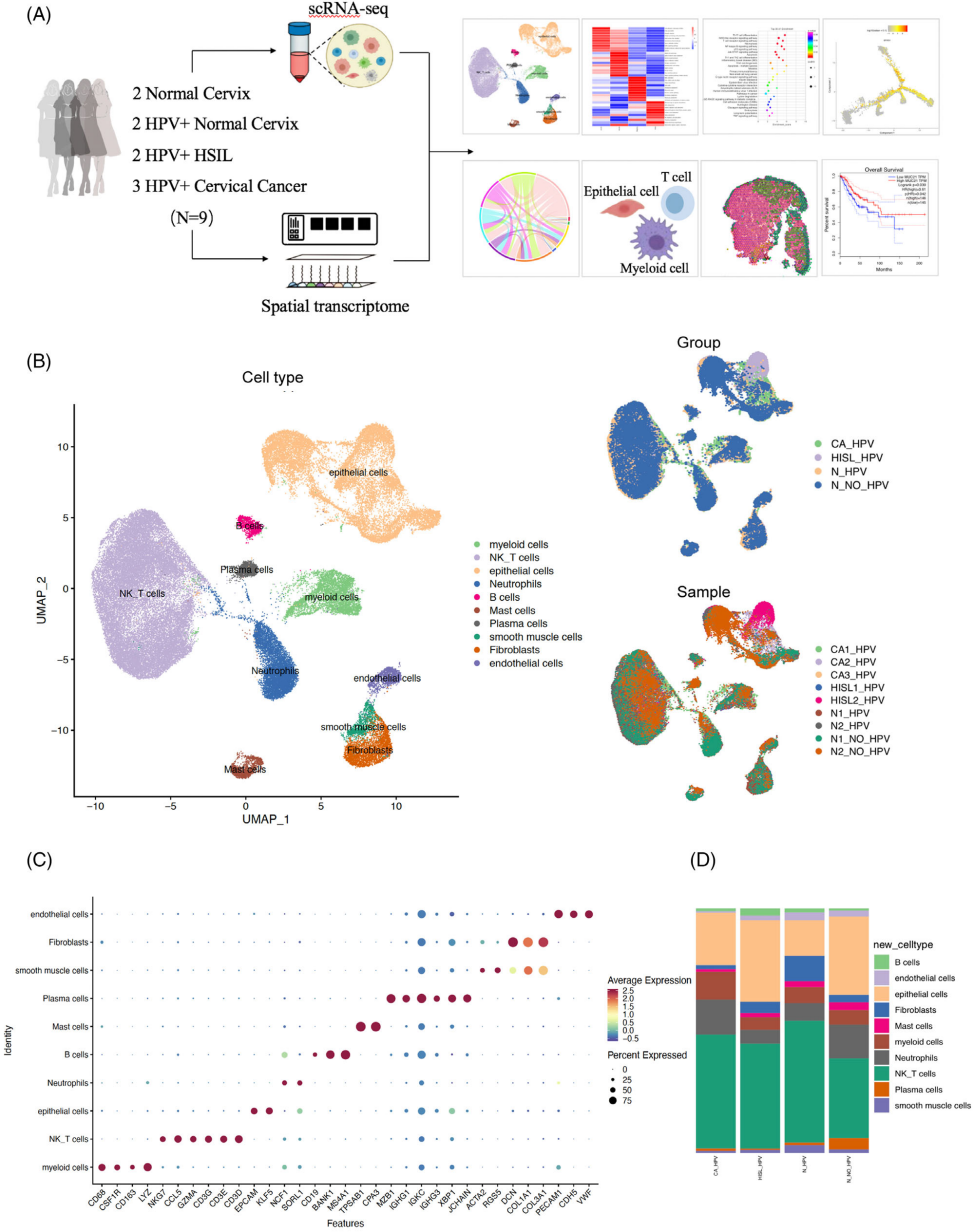
Single‐cell profiling of normal cervix, precursor lesions and cervical cancer: (A) overall flow chart of this present study; (B) the UMAP plot, showing cell types (left), clinical groups (upper right) and sample origin (lower right) of the cells captured by single‐cell RNA‐sequencing (scRNA‐seq); (C) the dotplot demonstrating the marker gene expression of each cell type including NK T cells, epithelial cells, neutrophils, myeloid cells, fibroblasts, mast cells, endothelial cells, plasma cells, smooth muscle cells and B cells; (D) the proportion of different cell types in four clinical groups, including normal cervix without human papillomavirus (HPV) infection, HPV‐infected normal cervix, HPV‐infected high‐grade squamous intraepithelial lesions (HSIL) and HPV‐infected cervical cancer.

As the overall workflow shows (Figure 1A), we first generated scRNA‐seq profiles from nine patient samples, including two HPV‐negative normal, two HPV‐positive normal, two HPV‐positive HSIL and three HPV‐positive cancer samples. After the initial quality control step, we acquired 5510–15 372 cells with 3547–12 940 unique molecular identifiers and 1334–3142 uniquely expressed genes per cell. To infer cell types, we generated a U‐MAP plot with projected marker gene expression (Figure 1B). There were 10 major cell types, 4 of which were structural cells, including epithelial cells (15 915 cells, 24%, marked with EPCAM, KLF5), fibroblasts (3221 cells, 4.9%, marked with DCN, COL1A1, COL3A1), endothelial cells (1317 cells, 2%, marked with PECAM1, CDH5, VWF) and smooth muscle cells (1129 cells, 1.7%, marked with ACTA2, RGS5). The other six cell types were immune cells, including myeloid cells (5078 cells, 7.6%, marked with CD68, CSF1R, CD163, LYZ), NK/T cells (28 784 cells, 43.5%, marked with NKG7, CCL5, GZMA, CD3G, CD3E, CD3D), neutrophils (7133 cells, 10.8%, marked with NCF1, SORL1), B cells (1008 cells, 1.5%, marked with CD19, BANK1, MS4A1), mast cells (1396 cells, 2.1%, marked with TPSAB1, CPA3) and plasma cells (1197 cells, 1.8%, marked with MZB1, IGHG1, IGKC, IGHG3, XBP1, JCHAIN) (Figure 1C). We found that epithelial cells were the most abundant structural cells, whereas NK/T and myeloid cells comprised most of the immune microenvironment (Figure 1D). In the subsequent analysis, we focused on these three major cell types to depict a comprehensive single‐cell transition landscape based on different stages from the normal cervix to precursor lesion to CC with persistent HPV infection.

### 2.2 HPV‐related epithelial subtypes: Specific features are altered during malignant progression from the normal cervix to precursor lesion to cancer

**FIGURE 2:**
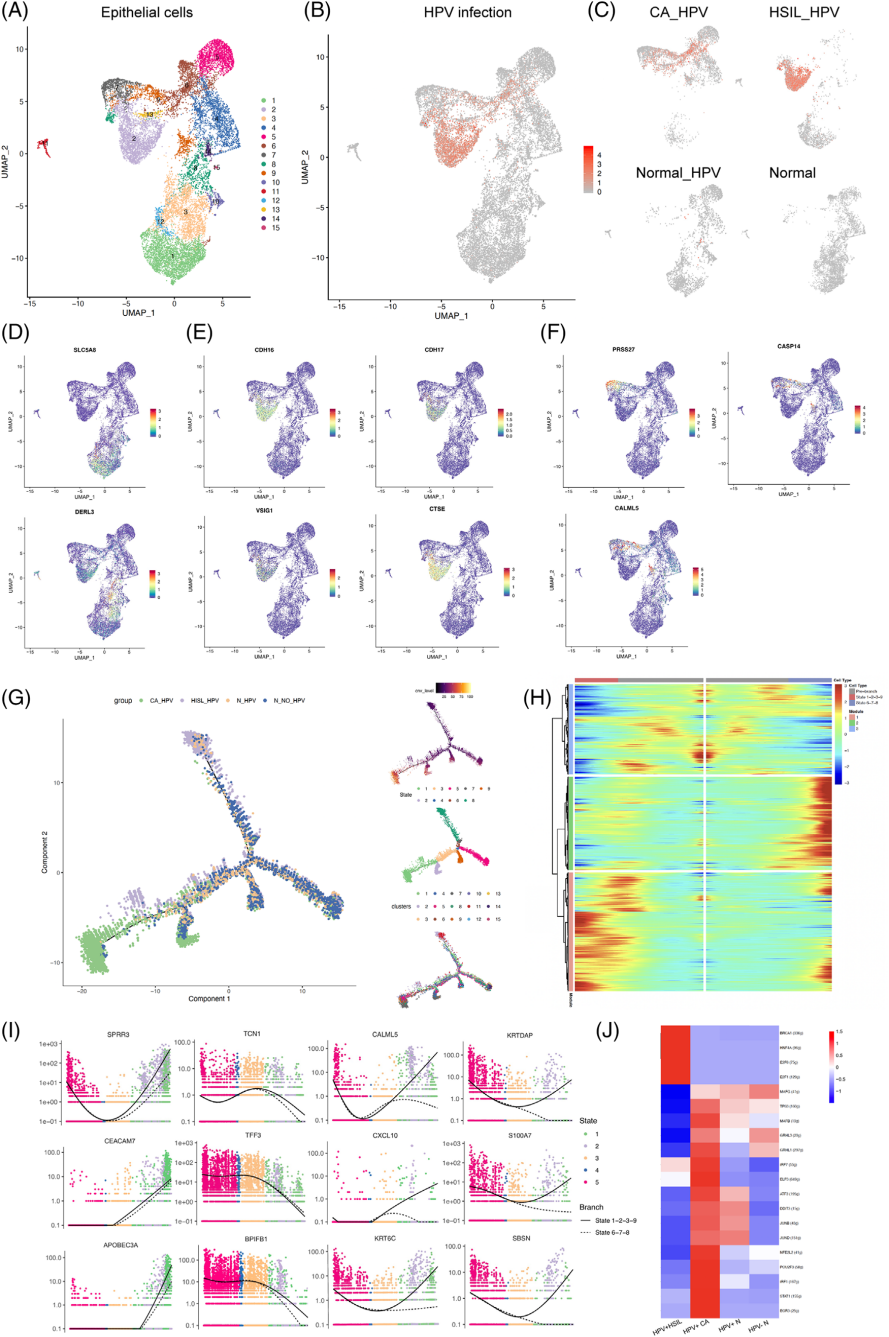
Human papillomavirus (HPV)‐related epithelial subtypes: Specific features are altered during malignant progression from the normal cervix to precursor to cancer: (A) the UMAP plot of the epithelial cells in 15 clusters; (B) the UMAP plot of the epithelial cells indicating HPV‐infected cells; (C) the UMAP plot of the epithelial cells indicating HPV‐infected cells within the 4 clinical groups, respectively, including normal cervix without HPV infection, HPV‐infected normal cervix, HPV‐infected high‐grade squamous intraepithelial lesions (HSIL) and HPV‐infected cervical cancer; (D) the UMAP feature plots of the marker genes of ‘HPV‐related‐normal cluster’ (SLC5A8 and DERL3); (E) the UMAP feature plots of the marker genes of ‘HPV‐related‐HSIL cluster’ (CDH16, CDH17, VSIG1 and CTSE); (F) the UMAP feature plots of the marker genes of ‘HPV‐related CA cluster’ (PRSS27, CASP14 and CALML5); (G) the pseudotime plots describing the differentiation trajectories of epithelial cells in the 4 clinical groups (left), with different CNV levels (upper right), with different inferred states (middle right) and in different clusters (lower right); (H) the beam analysis indicating the transition of gene expression during the transition from States 1‐2‐3‐9 to 6‐7‐8; (I) the node gene expression along the differential trajectories (SPRR3, CEACAM7, APOBEC3A, TCN1, TFF3, BPIFB1, CALML5, CXCL10, KRT6C, KRTDAP, S100A7 and SBSN); (J) the heat map of SCENIC analysis indicating the major transcriptional factors of epithelial cells in the 4 clinical groups, including normal cervix without HPV infection, HPV‐infected normal cervix, HPV‐infected HSIL and HPV‐infected cervical cancer.

Given the critical biological roles of epithelial cells in the TME of CC, we performed unsupervised clustering and acquired 15 clusters (Figure 2A). On this basis, considering the impact of HPV infection on malignant epithelial transformation, we performed HPV sequence blasting and identified 7 HPV‐infected epithelial clusters (1, 2, 3, 6, 7, 9, 13) (Figure 2B) (Figure S1). Notably, we observed that clusters 1 and 3 were mainly enriched in the normal group, cluster 2 was remarkably clustered in HSIL, and clusters 6, 7, 9 and 13 were prominent in CC (Figure 2C). Moreover, by analysing the marker gene expression and functional pathways of these ‘HPV‐related clusters,’ we discovered that clusters 1 and 3 enriched in the normal cervix featured antineoplastic effects and highly expressed multiple tumour suppressors (SLC5A8, DERL3) (Figure 2D), thereby suppressing cell proliferative, migratory and invasive capacities.^9^, ^10^ On the other hand, cluster 2, representing HSIL, manifested high cellular motor capacity, specifically expressing genes related to cell adhesion (CDH16, CDH17 and VSIG1)^11^, ^12^ and extracellular matrix degradation (CTSE) (Figure 2E), thus potentially promoting the expansion of atypical cells in intraepithelial neoplasia progression. Additionally, we observed that clusters 6, 7, 9 and 13 (CASP14, PRSS27, CALML5) (Figure 2F), which were enriched in CC, manifested high expression levels of genes related to carcinogenic pathways such as epithelial‐to‐mesenchymal transition, tumour cell proliferation, migration, invasion and angiogenesis. Further inferCNV analyses were conducted to confirm the malignancy of certain epithelial clusters. First, we designated all cells except epithelial cells as normal cells, and results showed that some epithelial cells had significant copy number variation compared with normal cells (Figure S1c). Then, in order to better explore the heterogeneity of tumour cells, we mapped the results of inferCNV into 15 clusters of epithelial cells. It was found that the degree of copy number variation of clusters 6,7,9 and 13 was significantly higher than that of other epithelial clusters (Figure S1d), and these clusters were uniquely located in cancer group rather than normal or precancer group (Figure S1e), which is of great significance to explore. Therefore, given the unique distribution, molecular features and function of the above ‘HPV‐related clusters’ in different stages across the progression process, we innovatively named three group‐specific ‘HPV‐related clusters.’ That is, we identified clusters 1 and 3 as ‘HPV‐related normal clusters,’ cluster 2 as ‘HPV‐related‐HSIL clusters’ and clusters 6, 7, 9 and 13 as ‘HPV‐related‐CA clusters.’ Overall, we discovered distinct features of HPV‐related epithelial cells, including antitumour effects in the normal cervix, increased cell motility in HSIL and enrichment of carcinogenesis pathways in CC, corresponding to the three ‘HPV‐related clusters.’ Collectively, these results vividly displayed the HPV‐induced malignant transition of epithelial cells.

To further investigate the malignant transformation among epithelial clusters from the perspective of cell fate and differentiation trajectories, we conducted pseudotime analysis (Figure 2G, Figure S3). Critically, the differentiation trajectory began with HPV‐negative normal cells that gradually transformed into HPV‐positive cells and terminated in two subsets, either HSIL or CA cells (Figure 2H). As the HPV‐infected cervix gradually developed precancerous lesions, we noticed six significant node genes (Figure 2I). Three (SPRR3, CEACAM7, APOBEC3A) might stimulate cervical lesion progression via a second hit after HPV infection.^13^, ^14^, ^15^, ^16^ The other three genes (TCN1, TFF3, BPIFB1) were downregulated in HSIL and CA compared with the normal cervix, revealing potentially weakened protection from malignant transition.^17^, ^18^, ^19^ These discoveries indicate that upregulation of those ‘second‐hit’‐related genes or downregulation of progression‐protective genes might initiate precancerous lesions and even CC. Thus, active therapy should be performed to prevent the malignant transition of cervical epithelial cells. Importantly, at the intersection of HSIL/CA, we identified six critical node genes (CALML5, CXCL10, KRT6C, KRTDAP, S100A7, SBSN) with great differentiation potential towards CC instead of HSIL,^20^, ^21^, ^22^, ^23^, ^24^ providing a valuable time window for early intervention at the precancer stage of CC. Furthermore, we next conducted SCENIC analysis to identify the decisive regulators of the epithelial subsets between HSIL and CA (Figure 2J). Strikingly, BRCA1,^25^ which play a crucial role in DNA replication, DNA repair and genomic stability maintenance,^26^ predominantly regulates the HPV‐related HSIL cluster, whereas TP63, ELF3 and POU2F3 modulate the abnormal proliferation and differentiation of epithelial cells in the HPV‐related CA cluster.^27^, ^28^ Overall, we creatively discovered 12 critical node genes and 5 regulons during the progression from the HPV‐infected normal cervix to HSIL to CA. These findings could facilitate accurate molecular subtyping and provide novel insights for the precise intervention of cervical lesions and tumours in clinical practice.

### 2.3 Higher immune‐suppressive potential coupled with active dysfunction of T cells in cervical cancer compared to precancer

**FIGURE 3:**
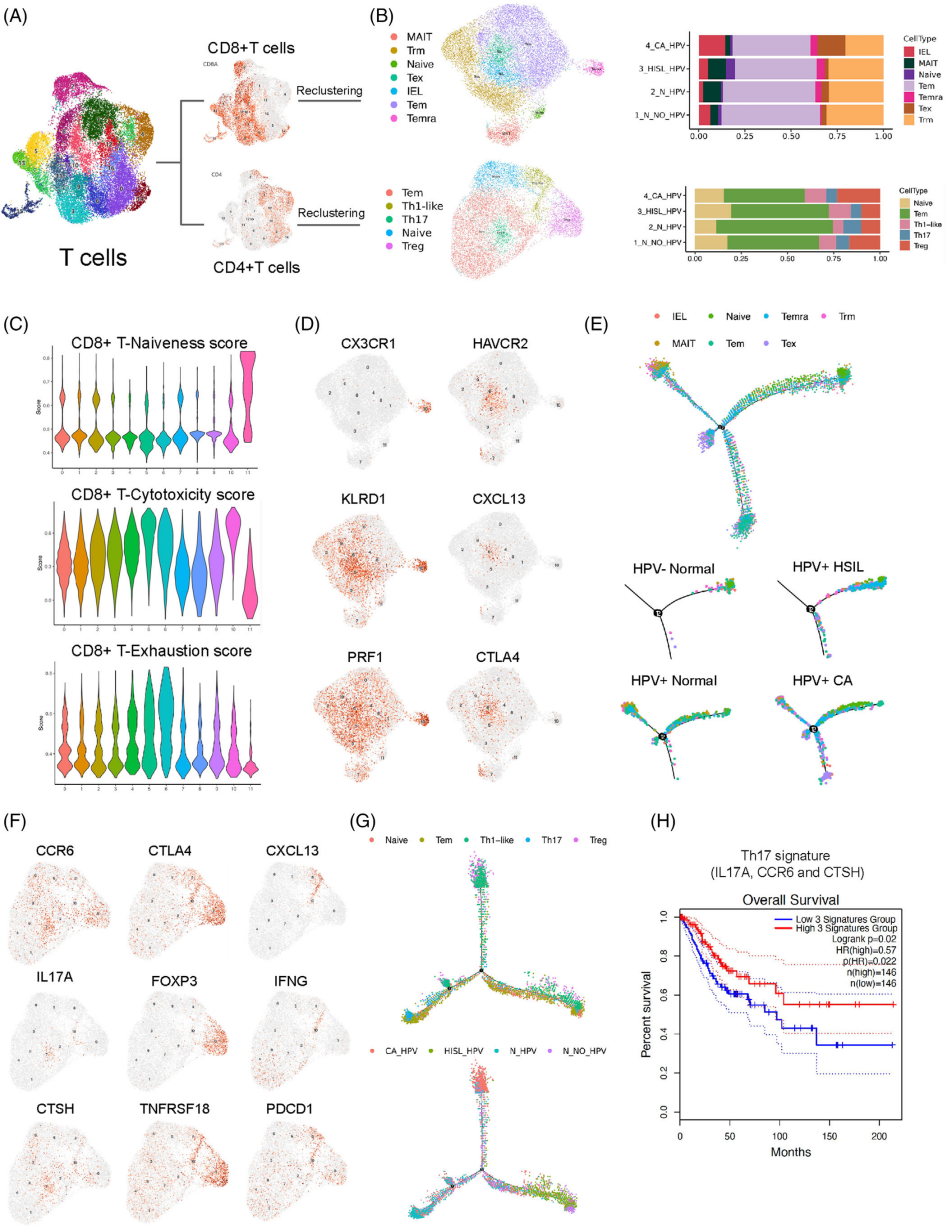
Higher immune‐suppressive potential coupled with active dysfunction of T cells in cervical cancer compared to precancer: (A) the UMAP plot of T lymphocytes which could be further reclustered into CD8+ T cells and CD4+ T cells; (B) the UMAP plot of the CD8+ T cells reclustered into seven subsets, including CD8+ MAIT, CD8+ Trm, CD8+ Naive, CD8+ Tex, CD8+ intraepithelial lymphocytes (IEL), CD8+ Tem and CD8+ Temra (upper left); the UMAP plot of the CD4+ T cells reclustered into five subsets, including CD4+ Tem, CD4+ Th1‐like, CD4+ Th17, CD4+ Naive and CD4+ Treg (lower left); the proportion of different CD8+ T cell subsets (upper right) and CD4+ T cell subsets (lower right), respectively, in four clinical groups, including normal cervix without human papillomavirus (HPV) infection, HPV‐infected normal cervix, HPV‐infected high‐grade squamous intraepithelial lesions (HSIL) and HPV‐infected cervical cancer; (C) the violin plots indicating the naiveness score (upper), cytotoxicity score (middle) and exhaustion score (lower) of each CD8+ T cell cluster; (D) the UMAP feature plots of the marker genes of CD8+ Temra (CX3CR1, KLRD1, PRF1) and CD8+ Tex (HAVCR2, CXCL13, CTLA4). (E) The pseudotime plot describing the differentiation trajectories of different subsets of CD8+ T lymphocytes (upper) and trajectory presented in the four clinical groups, respectively, including normal cervix without HPV infection, HPV‐infected normal cervix, HPV‐infected HSIL and HPV‐infected cervical cancer; (F) the UMAP feature plots of the marker genes of CD4+ Th17 (CCR6, IL17A, CTSH), CD4+ Treg (CTLA4, FOXP3, TNFRSF18) and CD4+ Th1‐like (CXCL13, IFNG, PDCD1); (G) the pseudotime plot describing the differentiation trajectories of different subsets of CD4+ T lymphocytes (upper) and trajectory presented based on the four clinical groups, including normal cervix without HPV infection, HPV‐infected normal cervix, HPV‐infected HSIL and HPV‐infected cervical cancer; (H) the Kaplan Meier curve predicting the overall survival (OS) of cervical cancer (CC) patients with high and low expression of the Th17 signature (IL17A, CCR6 and CTSH) using TCGA data (*p* = .02, log rank test).

As a crucial component and the most prevalent immune cell type in the TME, T cells were investigated and further grouped into 16 clusters according to unsupervised clustering (Figure 3A). Based on the function‐associated genes, the 16 clusters were classified into 7 CD8+ T‐cell subtypes and 5 CD4+ T‐cell subtypes (Figure 3B). With regard to CD8+ T cells, effector memory (Tem, clusters 0, 1, 4, 8, 9; *GZMK, CXCR4, CST7*), tissue‐resident memory (Trm, clusters 2, 3; *XCL1, CAPG, NR4A1*), intraepithelial lymphocytes (IEL, cluster 5; *CD160, KIR2DL4, KLRC2*), exhausted (Tex, cluster 6; *HAVCR2, CXCL13, PDCD1*), mucosa‐associated invariant (MAIT, cluster 7; *SLC4A10, KLRB1, ZBTB16*), recently activated effector memory (TemRA, cluster 10; *CX3CR1, FCGR3A, FGFBP2*) and naïve (Tn, cluster 11; *LEF1, CCR7, SELL*) CD8+ T cells were identified (Figure S2). To reveal the functional status of the different CD8+ T‐cell subsets, we classified these CD8+ T‐cell subsets based on the expression of genes associated with naïve, cytotoxic and exhausted phenotypes (Figure 3C). As expected, the Tn cluster tended to be nonactivated, whereas Temra, featuring high expression of CX3CR1, presented a high cytotoxicity signature (*PRF1, KLRD1*), which was reported to be involved in fighting against persistent viral infection and infiltration into tumour regions with high cytotoxicity and migratory abilities.^29^, ^30^ These findings demonstrate a positive antitumour immune function of CD8+ CX3CR1+ Temra. In addition, Tex cells highly expressed *HAVCR2, CXCL13, PDCD1* and *CTLA4*, which demonstrated the dysregulation and exhaustion of CD8+ Tex cells (Figure 3D). Furthermore, to explore the transformation within different T‐cell subsets in the functional state, pseudotime analysis and RNA velocity were applied to diverse CD8+ cell subtypes (Figure 3E, Figure S4). We observed a differentiation trajectory initiated with a naïve cluster that gradually transitioned to Tem and TemRA, eventually ending in Tex. Moreover, MAIT cells, IELs and Trm cells, which play a significant role in immune surveillance, were ubiquitous along the whole differentiation trajectory of CD8+ T cells.

Having investigated the gene expression pattern and differentiation trajectories of the above CD8+ T‐cell clusters, we further explored the discrepancy in abundance and function among various CD8+ T‐cell subsets among four groups (HPV‐negative normal cervix, HPV‐positive normal cervix, HPV‐positive HSIL and HPV‐positive CA) during the malignant transition process. Compared to the precancer groups, the total CD8+ T‐cell levels were elevated in the CA group. Notably, CD8+ Tex was more abundant in CA, whereas the MAIT cluster was prominent in HSIL, potentially due to its immune surveillance function in the precancerous stage. In addition, we unexpectedly found that TemRA abundance was elevated after HPV infection, was highest when lesions progressed to HSIL and was slightly decreased in CA, indicating its antiviral role and further transition to Tex when lesions escalated. Furthermore, we calculated exhaustion/cytotoxicity scores among the four groups and found that the score increased as the disease stage progressed. These results indicated a shift to an immune‐suppressive microenvironment across the progressive course from normal to precursor lesion to cancer. In general, the CD8+ T‐cell subtype was altered and exhibited a progressively immunosuppressive trajectory during the malignant transition from normal to precursor lesion to cancer, during which the abundance of CD8+ Temra exhibiting a positive immune reaction gradually decreased, and the infiltration of CD8+ Tex cells demonstrating immune dysfunction propelled the malignant program.

Next, we focused on CD4+ T cells and identified five subtypes (Figure 3F, Figure S2), including Tregs (cluster 0; FOXP3, CTLA4, TNFRSF18), Tems (clusters 1, 2, 3, 4, 9; CCL5, GZMK, CXCR4), naïve T cells (clusters 5, 6; CCR7, SELL, LEF1), Th17 T cells (cluster 7; IL17A, CCR6, CTSH) and Th1‐like T cells (clusters 8, 10; CXCL13, PDCD1, IFNG). Notably, Th17 cells expanded after HPV infection and decreased in number as cervical lesions developed. In addition, marked infiltration of Th1‐like and Treg cells was observed during the progression from HPV infection to precursor lesion to CC. In addition, Th1‐like cells greatly expanded as persistent HPV infection progressed into HSIL, whereas Treg cells dominated the immune microenvironment during the malignant transition from HSIL to cancer. Further pseudotime analysis and RNA velocity exhibited an evolutionary path initiated with naïve CD4+ T cells that differentiated into Tem or Th17 cells and ended with Treg and Th1‐like CD4+ T cells, further verifying the potential plasticity of Th17 cells to become Th1‐like or Treg cells (Figure 3G, Figure S4). Based on the previous findings, we speculated that Th17 cells might promote cervical barrier defence and immunity against pathogens early in HPV infection. Additionally, the loss of Th17/Treg homeostasis might lead to the failure of HPV clearance and disease progression, whereas an imbalanced Th1‐like/Treg axis might lead to carcinogenesis due to the shift from an inflammatory niche to an immunosuppressive environment in CA. Notably, a survival analysis using TCGA data showed that Th17‐cell (IL17A+, CCR6+, CTSH+) abundance correlated with better CC patient OS rates (Figure 3H), highlighting the antiviral properties of Th17 cells and the potential for targeting these cells for early intervention in CC. In summary, the transition and imbalance among different subsets of CD4+ T cells, especially Th17, Th1‐like and Treg cells, could promote the transformation from antiviral immune responses to persistent HPV infection, then to precancerous lesions and, ultimately, CC.

### 2.4 Potential targets for distinguishing cancer from precancer within the myeloid lineage: pDCs and cycling cells

**FIGURE 4:**
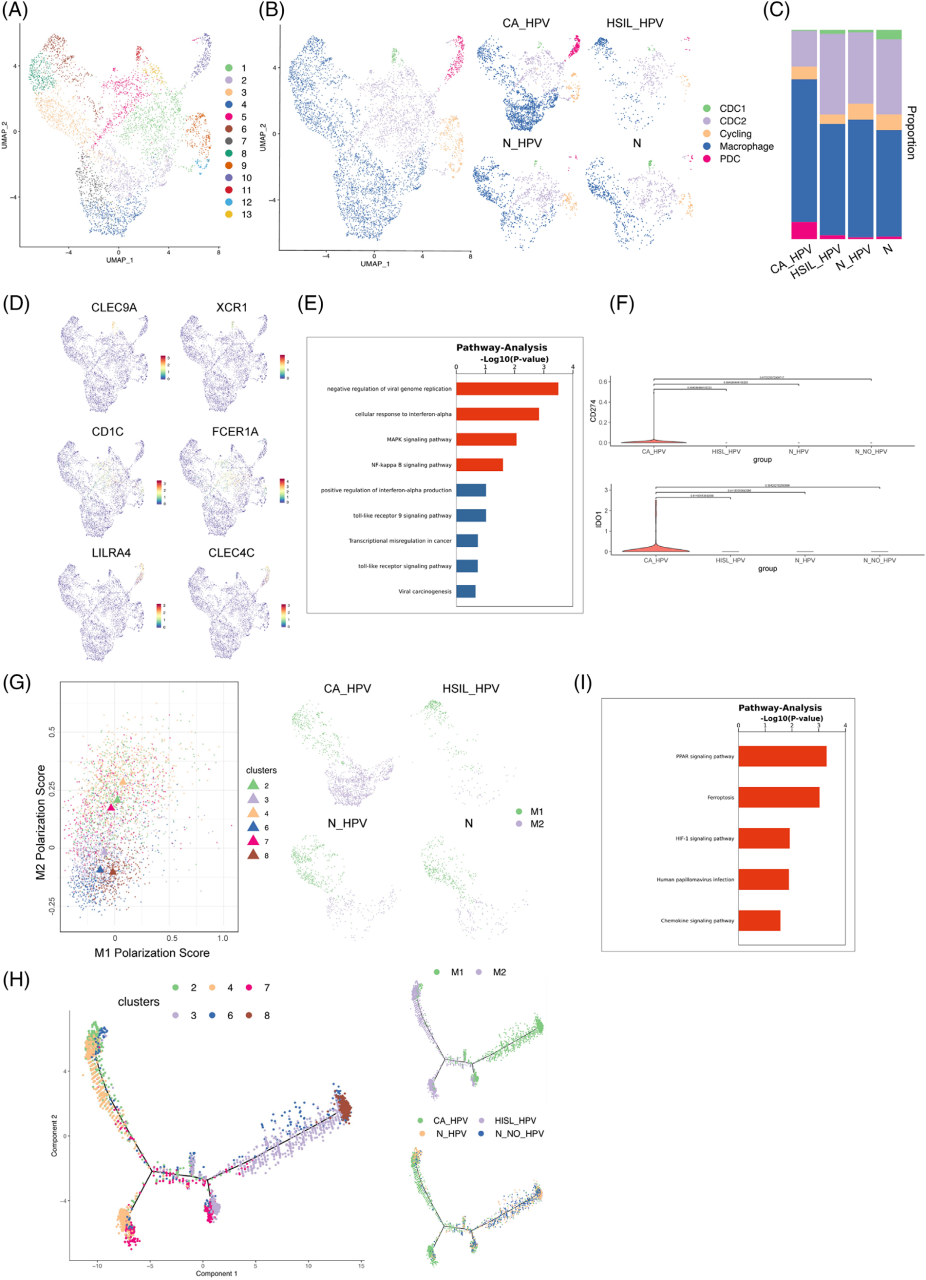
Potential targets for distinguishing cancer from precancer within the myeloid lineage: plasmacytoid DCs (pDCs) and macrophages: (A) the UMAP plot of the myeloid cells reclustered into 13 clusters; (B) the UMAP plot of the myeloid cells categorized into 5 subsets, including CDC1 cells, CDC2 cells, cycling cells, macrophages and pDCs (left); the UMAP plots of the myeloid cells presented in the 4 clinical groups, respectively, including human papillomavirus (HPV)‐infected cervical cancer, HPV‐infected high‐grade squamous intraepithelial lesions (HSIL), HPV‐infected normal cervix and normal cervix without HPV infection (right); (C) the proportion of different myeloid cell subsets in the 4 clinical groups; (D) the UMAP feature plots of the marker genes of CDC1 cells (CLEC9A, XCR1), CDC2 cells (CD1C, FCER1A) and pDCs (LILRA4, CLEC4C); (E) the bar plot indicating the functional pathways of pDCs through Pathway analysis; (F) the violin plots manifesting the expression of IDO1 (upper) and CD274 (lower) in pDCs of the 4 clinical groups; (G) the M1/M2 polarization score of the macrophages in clusters 2, 3, 4, 6, 7 and 8 (left); the UMAP plot of the macrophages exhibiting the M1/M2 polarization state in the 4 clinical groups, respectively; (H) the pseudotime plot elucidating the differentiation trajectories of different clusters of macrophages (left) and trajectory presented in M1/M2 polarization state (upper right) and according to the 4 clinical groups (lower right); (I) the bar plot indicating the functional pathways of macrophages in clusters 2, 4 and 7 through pathway analysis.

We performed U‐MAP clustering on 5078 myeloid cells to investigate the variation in specialized myeloid cell populations within the immune microenvironment during the malignant progression from precancer to CC and identified 13 clusters (Figure 4A, Figure S2). These 13 clusters were further regrouped into 5 major cell types based on functional markers (Figure 4B,C), including cDC1 (cluster 11), cDC2 (clusters 1, 5 and 13), plasmacytoid DCs (pDCs, cluster 10), macrophages (clusters 2, 3, 4, 6, 7, 8) and cycling cells (clusters 9, 12). We first studied DCs, as these cells are crucial components that induce and maintain antitumour immunity in the TME. Three distinct subsets of DCs were identified (Figure 4D), namely cDC1 (XCR1+/CLEC9A+), cDC2 (CD207+/CD1C+/FCER1A+) and pDC (CLEC4C+/LILRA4+). Further functional pathway analysis showed that pDCs might be activated by Toll‐like receptor 7/9 (Figure 4E). Additionally, pDCs modulate immune responses to viral infection by producing IFN‐α and negatively regulate viral genome replication in the early HPV infection stage.^31^ However, as the virus infection persisted, pDCs markedly accumulated in the CA. Notably, we observed decreased IFN‐α production and increased expression of some immunosuppressive mediators (IDO1 and PD‐L1) in the CA group (Figure 4F) (Figure S5). Surprisingly, we found that the MAPK and NF‐kappa B pathways were enriched in CA‐derived pDCs, implying that TA‐pDCs (tumour‐associated pDCs) exert oncogenic effects during chronic viral infection. Overall, these results indicate that the functional impairment of pDCs might lead to the failure of virus clearance, immune tolerance and even immune escape. Significantly, promoting IFN‐α‐induced antiviral responses and eradicating functionally impaired pDCs are potential novel pDC‐targeted therapies for persistent HPV infection and CC.

Activated macrophages are classified into proinflammatory (M1‐like) or anti‐inflammatory (M2‐like) subtypes based on their responses to different microenvironmental stimuli. We calculated the M1 and M2 polarization scores using related gene sets to depict the M1/M2 signature of macrophages and understand the mechanisms that control the repertoire of macrophage phenotypes during the malignant transition from precancer to cancer (Figure 4G). The results showed that among the 6 macrophage cell clusters (2, 3, 4, 6, 7, 8), clusters 3, 6 and 8 were more inclined to have an M1/proinflammatory status, whereas clusters 2, 4 and 7 exhibited higher M2/anti‐inflammatory scores with overexpression of *CD163* and *IL1RN*. Pseudotime trajectory analysis further verified the M1‐ to M2‐phenotype shift as the disease escalated (Figure 4H). The subsequent pathway enrichment analysis showed that macrophages in clusters 2, 4 and 7 were involved in PPAR, interleukin‐10 and HIF‐1α signalling, which were reported to activate M2 polarization, inhibit cytokine production and shape a hypoxic immunosuppressive TME,^32^, ^33^ respectively, thus exerting protumour effects (Figure 4I). Accordingly, a shift from the M1 to the M2‐like polarization phenotype played a pivotal role in the qualitative change from precancer to CA, highlighting macrophage polarization as a potential treatment target in CC.

### 2.5 Cell–cell communication among epithelial, T and myeloid cells in the CC immune microenvironment

**FIGURE 5:**
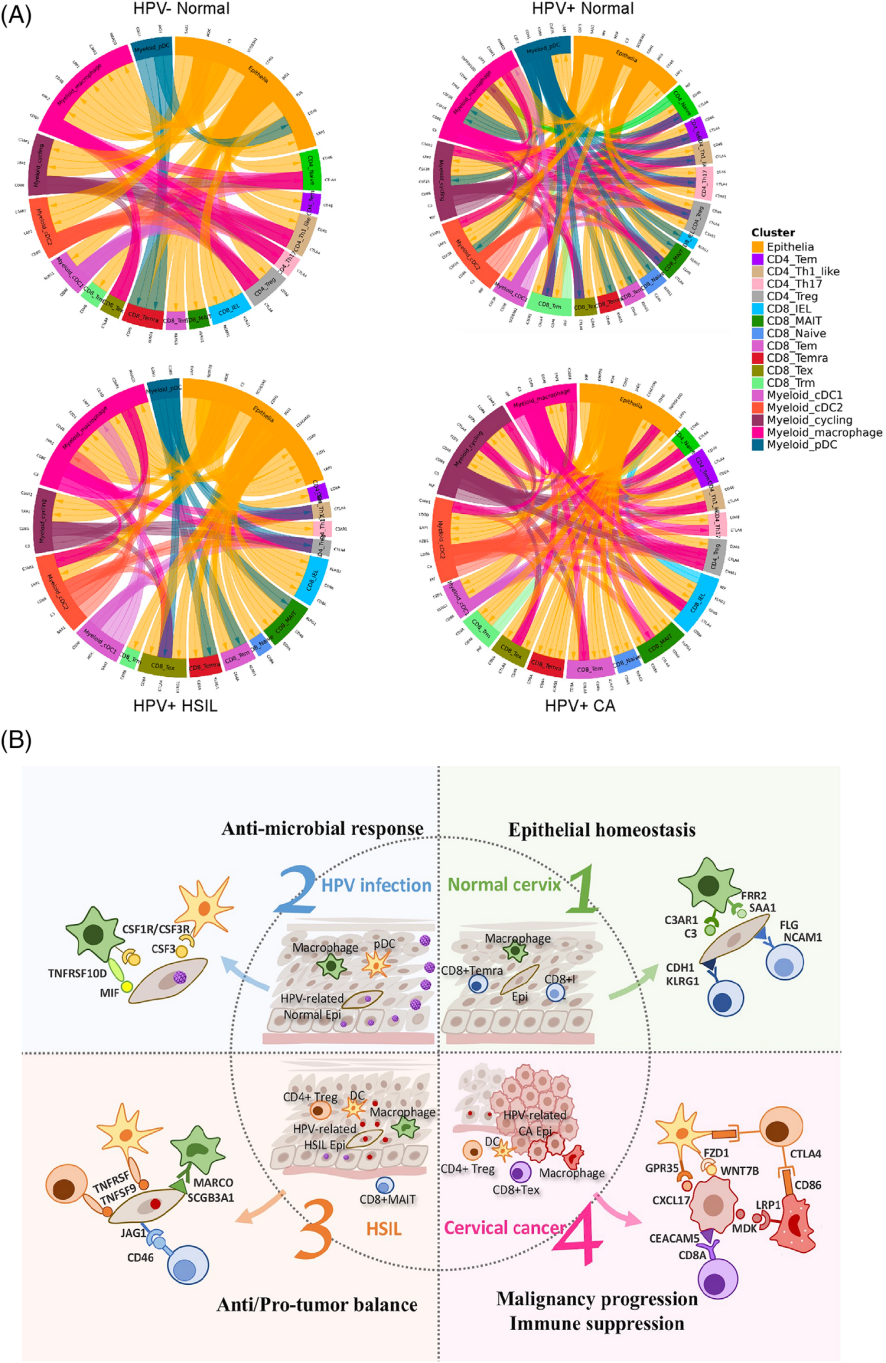
Cell–cell communication among epithelial, T and myeloid cells in the cervical cancer (CC) immune microenvironment: (A) the circos plots describing cellular interaction among epithelial, T lymphocytes and myeloid cells in the four clinical groups, respectively, including normal cervix without human papillomavirus (HPV) infection, HPV‐infected normal cervix, HPV‐infected high‐grade squamous intraepithelial lesions (HSIL) and HPV‐infected cervical cancer; (B) the scheme demonstrating detailed ligand–receptor pairs, respectively, mediating the epithelial homeostasis in normal cervix, anti‐microbial response in HPV‐infected cervix, anti/protumour balance in HSIL and malignancy progression in cervical cancer.

We conducted CellphoneDB analysis using our high‐resolution scRNA‐seq data to reveal intercellular communication among epithelial, T and myeloid cells (Figure 5A). Strikingly, we discovered unique cellular crosstalk in different disease stages, including HPV‐negative normal cervix, HPV‐positive normal cervix, HPV‐positive HSIL and HPV‐positive CC. Specifically, (1) in the normal cervix without HPV infection, we noticed ligand–receptor pairs such as FLG–NCAM1, C3–C3AR1, CDH1–KLRG1 and SAA1–FPR2, which were responsible for the maintenance of normal epithelial function regarding immune surveillance, injury repair and anti‐inflammation.^34^ (2) When cervical tissue was infected with HPV, the MIF–TNFRSF10D interaction was enhanced, demonstrating anti‐inflammatory and antiviral effects. Additionally, we observed a CSF3–CSF1R/CSF3R interaction that regulated macrophage differentiation and stimulated the immune response in HPV‐infected cervical tissue.^35^ (3) As persistent HPV infection gradually resulted in the transformation of cervical tissue into HSIL, the antitumour and protumour effects converged, and the former appeared more remarkable. Specifically, the SCGB3A1–MARCO interaction exerted an anti‐microbial effect, whereas the extensive TNFSF9–TNFRSF crosstalk between epithelial cells and immune cells potentially contributed to macrophage M1 polarization and inhibited the invasion and migration of HPV‐infected epithelial cells.^36^ These ligand–receptor interactions probably helped prevent HSIL from progressing to CC. Moreover, the JAG1–CD46 interaction was detected in only HSIL and not at other disease stages; this interaction has been reported to induce the differentiation of CD4+ T cells into Tregs, representing the emergence of immunoregulatory features. (4) As precancerous lesions progressed into CC, we discovered ligand–receptor interactions, including CXCL17–GPR35 and WNT7B–FZD1, which exerted protumour effects by stimulating cancer cell proliferation and migration.^37^, ^38^, ^39^ In addition, we detected CEACAM5–CD1D interactions between HPV‐related CA cells and myeloid cells and CEACAM5–CD8A interactions between HPV‐related CA cells and CD8+ T cells. Given that CEACAM5 expression is closely related to HPV DNA integration into the host genome,^40^ we inferred that the above cellular crosstalk mediated tumour‐associated antigen presentation and immune activation of CD8+ T cells. On the other hand, multiple immunosuppressive cellular communications were dominant in the CC TME. For instance, CD86–CTLA4 interactions marked the immunoregulatory effects of CD4 Tregs within the tumour immune microenvironment, whereas MDK–LRP1 interactions promoted immune‐tolerant phenotypes in macrophages and induced CD8+ T‐cell dysfunction.^41^, ^42^ Overall, we intuitively depicted the cellular communication unique to the four disease stages, characterizing epithelial homeostasis in the normal cervix, antimicrobial immune response after HPV infection, the balance between antitumour and protumour effects in HSIL, and, last but not least, malignancy progression and immune suppression in CC.

Above all, our cellular interaction analysis sheds light on the mechanisms underlying the malignant transition from HPV infection to precancer to CC and reveals multiple potential novel therapeutic approaches for CC (Figure 5B), for example, anti‐CTLA4 therapy to prevent immune dysfunction and WNT7B inhibition to block cancer invasion and progression.

### 2.6 Spatial profiles of cell type subpopulations across tissue regions in the HPV‐infected normal cervix, precursor lesion and cervical cancer

**FIGURE 6:**
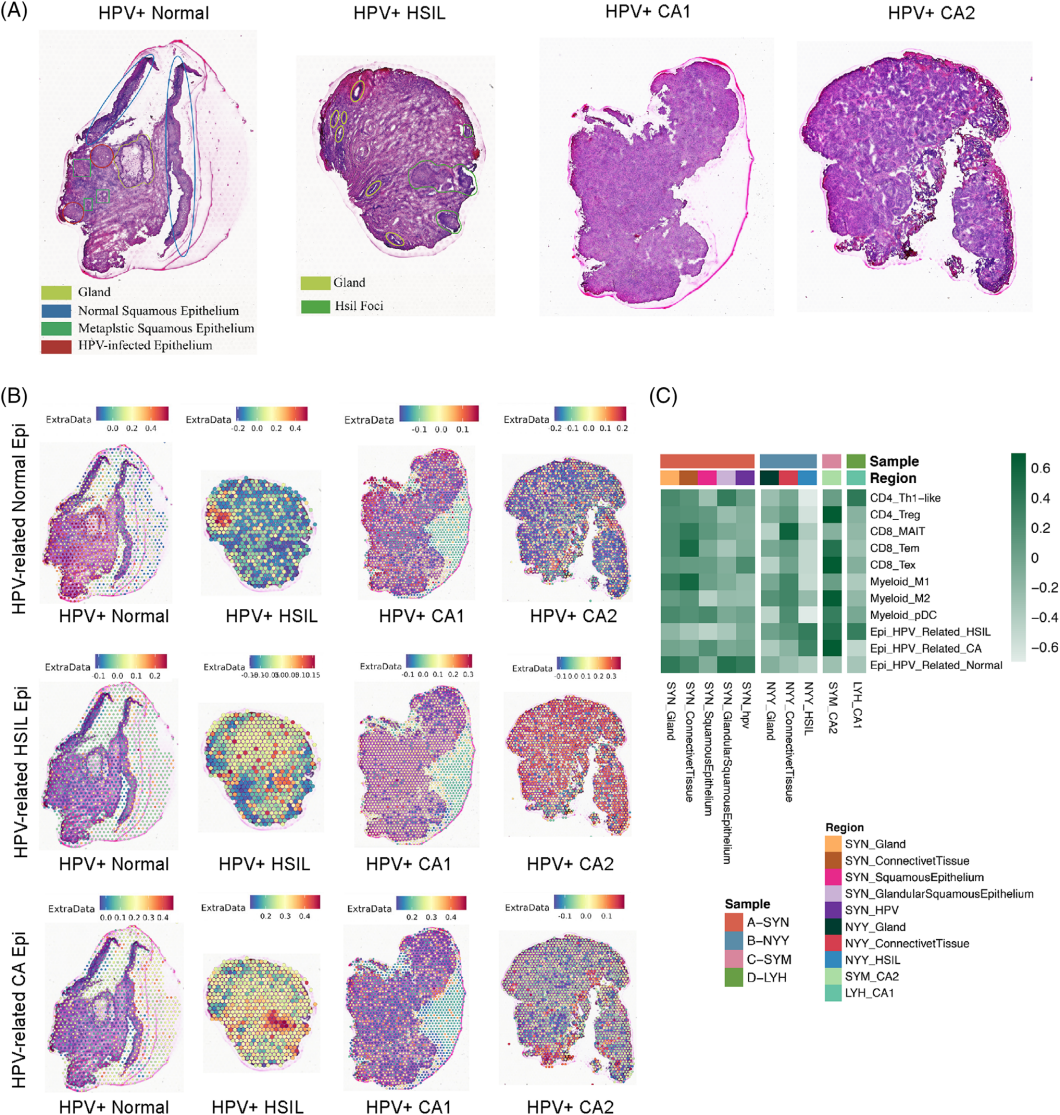
Spatial profiles of cell type subpopulations across tissue regions in the human papillomavirus (HPV)‐infected normal cervix, precursor lesion and cervical cancer. (A) HE slides of four clinical samples, including one HPV‐positive normal cervix (SYN), one HPV‐positive high‐grade squamous intraepithelial lesion (HSIL) (NYY) and two HPV‐positive cervical cancer tissues (SYM, LYH). Within the HE slide of HPV‐positive normal cervix, four specific areas were marked as gland, normal squamous epithelium, metaplastic squamous epithelium and HPV‐infected epithelium, whereas the remaining area was identified as connective tissues. Within the HE slide of HPV‐positive HSIL, two specific areas were marked as gland and HSIL foci, whereas the remaining area was identified as connective tissues. Within the HE slides of two HPV‐positive cervical cancer tissues, the whole area was recognized as cancer foci. (B) Comparison of the relative intensity of three HPV‐related epithelial clusters (upper: ‘HPV‐related Normal epithelial cluster,’ medium: ‘HPV‐related HSIL epithelial cluster’ and lower: ‘HPV‐related CA epithelial cluster’) among four clinical samples, including the HPV‐positive normal cervix (SYN), HPV‐positive HSIL (NYY) and two HPV‐positive cervical cancer tissues (SYM, LYH). (C) The heat map indicating the comparison of the relative intensity of three HPV‐related epithelial clusters, CD4+, CD8+ and myeloid cell subtype scores among different regions, including normal, precursor lesions and cervical cancer within four clinical samples. The expression of SYN was significant in HPV‐related normal epithelial clusters. The expression of NYY was significant in CD8+ MAIT cells, HPV‐related HSIL epithelial clusters. The expressions of SYM and LHY were significant in CD8+ Tex cells, CD4+ Tregs, myeloid M2, plasmacytoid DC (pDC) and HPV‐related CA epithelial clusters.

To study the spatial architecture of diverse cell subsets during the progression from HPV‐infected normal cervix tissues to precursor lesion to CC, we first generated transcriptomic maps by mounting cryosections of four cervical tissues originating from HPV‐positive normal cervix, HPV‐positive HSIL, HPV‐positive SCC and HPV‐positive AC onto spatially barcoded ST microarray slides. Then, distinct histological features were annotated after hematoxylin and eosin staining and brightfield imaging (Figure 6A). Specifically, eight regions were identified: gland, connective tissue, normal squamous epithelium, HPV‐infected epithelium, metaplastic squamous epithelium, HSIL foci and CC. The histological gene expression map revealed that epithelial marker genes were highly expressed in regions previously annotated as normal stratified squamous epithelium/glands or precancer/CC foci. In addition, marker genes for fibroblasts and endothelial cells were remarkably expressed in the regions annotated as connective tissues. These results verified the consistency of spatial gene expression patterns with the previous eight identifiable regions. Moreover, we observed that immune cells, including myeloid and T cells, generally surrounded epithelial cells, especially in CC, revealing the tumour immune microenvironment spatially.

We also investigated the locations of various cell subpopulations and unique tissue structures during different disease stages (Figure S6). HPV blasting analysis indicated that in the tissue of the HPV‐positive normal cervix, the titre of HPV infection was quite low in HPV‐infected epithelium, whereas in HPV‐positive HSIL tissue, HPV gene integration mainly occurred in HSIL foci instead of glands and connective tissue regions. Moreover, we discovered an extensive spatial distribution of HPV gene expression in SCC tissue, confirming the decisive contribution of persistent HPV infection to cervical carcinogenesis. Subsequently, we mapped the gene expression of the three previously innovatively identified ‘HPV‐related epithelial clusters’ onto ST slides (Figure 6B and Figure S7). (1) Of note, the ‘HPV‐related normal epithelial cluster’ showed great abundance in the HPV‐positive normal cervix sample, expressing SLC5A8 and DERL3, mainly in the HPV‐infected and metaplastic squamous epithelium, highlighting the spatial initiation of the HPV‐induced malignant transition. (2) In addition, the ‘HPV‐related HSIL epithelial cluster’ was prominent in the HPV‐positive HSIL tissue sample specifically within the HSIL foci, highly expressing VSIG1 and CASC9. (3) Furthermore, the ‘HPV‐related CA cluster’ was detected in the ST slides of CC, with high expression of CASP14 and CALML5. Based on these results, we innovatively named the three ‘HPV‐related epithelial cell clusters’ ‘SLC5A8+ DERL3+ HPV‐related normal epithelial cluster,’ ‘VSIG1+ CASC9+ HPV‐related HSIL epithelial cluster’ and ‘CASP14+ CALML5+ HPV‐related CA cluster.’ Additionally, based on the perfect reflection of the three unique ‘HPV‐related epithelial clusters,’ we identified the tissue architecture and provided a promising treatment strategy for targeting these unique cell clusters.

Having investigated the spatial location of HPV‐related epithelial cells, we then focused on the spatial profiles of myeloid cells and T lymphocytes (Figure 6C). Regarding CD8+ T cells, we discovered a greater infiltration of CD8+ Tex cells in CC. Intriguingly, we noticed a consistent spatial distribution of CD8+ Tex cells and HPV‐related CA epithelial clusters in CC tissues, demonstrating that the interaction of these two cell subsets potentially mediates CD8+ T‐cell dysfunction and tumour immune escape. Moreover, CD8+ MAIT cells showed great abundance in HSIL tissues, including glands, connective tissues and HSIL foci, playing a significant role in immune surveillance. Regarding CD4+ T cells, we found a greater abundance of CD4+ Tregs in CC tissues than in normal cervix and HSIL tissues, verifying the immunosuppressive feature of the CA microenvironment.

Considering the spatial localization of interacting cells, we evaluated the coexpression profiles of the previously discovered key ligand–receptor pairs during different disease stages using histological slides. (1) In the early phase of HPV infection, CSF3 (‘SLC5A8+ DERL3+ HPV‐related normal epithelial cluster’) and CSF1R/CSF3R (myeloid cells) were coexpressed within the HPV‐infected squamous epithelium and metaplastic squamous epithelium, which activated the immune response. (2) As HPV infection persisted, spatial coexpression of TNFSF10 (‘VSIG1+ CASC9+ HPV‐related HSIL epithelial cluster’) and TNFRSF10B (myeloid cells) was observed in the HSIL foci, which mediated the apoptosis of aberrant cells, revealing the immune surveillance and clearance of abnormal epithelial cells during the precancer stage. Moreover, within the same HSIL foci, marked JAG1 (‘VSIG1+ CASC9+ HPV‐related HSIL epithelial cluster’)‐CD46 (CD8+MAIT) coexpression, responsible for inhibitory immune regulation, was also observed. These results indicate that both positive and negative immune responses exist in HSIL, with the former holding a dominant position. (3) In the histological maps of CC, the spatial coexpression of ligand–receptor pairs, such as WNT7B (‘CASP14, CALML5+ HPV‐related CA cluster’)‐FZD1 (cDC), representing the aggressiveness of cancer, CEACAM5 (‘CASP14, CALML5+ HPV‐related CA cluster’)‐CD1D (cDC), manifesting tumour‐associated antigen presentation and TNFSF10 (‘CASP14, CALML5+ HPV‐related CA cluster’)‐TNFRSF11B (macrophages), embodying tumour escape through anti‐apoptosis, were remarkably discovered, fully demonstrating the malignant features of cancer. Collectively, these results clearly elucidate the spatial mapping of cellular interactions during distinct stages from HPV infection to precancerous lesions to CC and provide treatment targets with unprecedented potential at different disease stages.

In summary, for the first time, we integrated scRNA‐seq with ST analysis and spatiotemporally demonstrated the mechanism of HPV‐related CC carcinogenesis in both the malignant transition of epithelial cells and the remodelling of the immune microenvironment. Through comprehensive analysis of the gene expression patterns, differentiation trajectories, functional pathways, cellular crosstalk and spatial distribution of specific cell subsets, we discovered multiple promising therapeutic targets, representing tremendous clinical value in CC.

## 3 DISCUSSION

**FIGURE 7:**
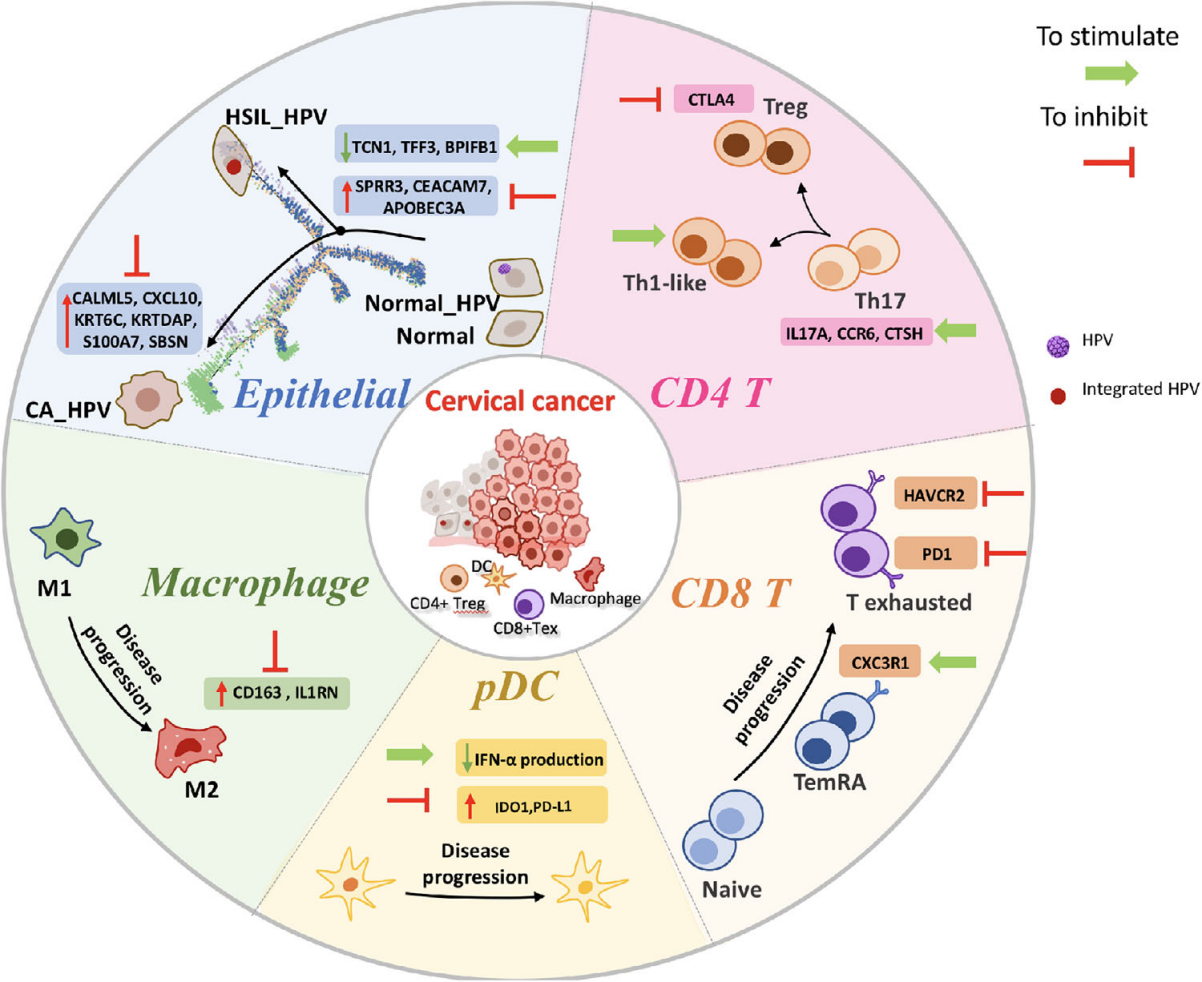
The schematic diagram demonstrating potential therapeutic targets (either to stimulate or to inhibit) regarding specific cell subsets such as epithelial cells, macrophages, plasmacytoid DCs (pDCs), CD4+ T cells and CD8+ T cells.

Persistent HPV infection is a crucial premise in cervical carcinogenesis; however, the exact cellular events, molecular virology and architecture transformation underlying the previous transition steps from normal cervical tissues and precursor lesions to CC remain elusive. (1) Are specific cell populations and molecules unique to different disease stages? Is there any specific cell differentiation trajectory? (2) What is the decisive factor that drives the transition from persistent HPV infection to precursor lesions and from HSIL to CC? (3) Why do some patients with persistent HPV infection gradually develop cancer, whereas others maintain normal status or even achieve HPV clearance? To answer these questions, for the first time, we integrated scRNA‐seq with ST analysis and depicted the cellular subset temporal transition and spatial location from normal cervical tissues (including both HPV‐infected and non‐HPV‐infected) to precancerous lesions to CC at the single‐cell level. Regarding cervical epithelial cells, we innovatively identified three unique clusters, including ‘SLC5A8+ DERL3+ HPV‐related normal,’ ‘VSIG1+ CASC9+ HPV‐related HSIL’ and ‘CASP14+ CALML5+ HPV‐related cancer.’ Excitingly, we discovered critical node genes that determined the cell fate between HPV infection and cervical lesions and cervical lesions and CC. Moreover, we observed a higher immune‐suppressive microenvironment in CC samples than in noncancer samples. Regarding CD4+ T cells, Treg/TH17 imbalance was associated with the change from persistent HPV infection to precancerous lesions, whereas Treg/TH1‐like imbalance was related to the progression from HSIL to CC. Regarding CD8+ T cells, more exhaustion and weaker cytotoxicity were observed in CC than in precancerous lesions. In addition, we found an M1–M2 transition of macrophages and pDC dysfunction during disease progression. Finally, the cellular interaction and ST combined analysis further verified distinct features in different disease stages, including epithelial homeostasis in the normal cervix, antimicrobial immune response after HPV infection, the balance between antitumour and protumour effects in HSIL, and finally, malignancy progression and immune suppression in CC. Our findings not only generated a comprehensive single‐cell and spatial atlas and uncovered cell trajectory fate along the cancerous process but also provided more possibilities for future advanced diagnostics and precise molecular therapies for CC (Figure 7).

For cervical epithelial cells, (1) we, for the first time, identified and creatively named three ‘HPV‐related clusters’ unique to distinct disease stages. Among them, the ‘HPV‐related normal cluster’ specifically expressed SLC5A8, a tumour suppressor gene, which has been reported to inhibit cell proliferation and regulate cell apoptosis.^9^, ^43^ This finding indicated that the protective mechanism would be activated accordingly after HPV infection. The precancerous lesion of CC, the ‘HPV‐related HSIL cluster,’ highly expressed VSIG1, which is also a biomarker of colorectal cancer precursor lesions.^12^ Therefore, we hypothesized that VSIG1 might be a critical molecule in precancerous epithelial lesions, which is worth further experimental investigation. Moreover, the ‘HPV‐related CA cluster’ specifically expressed CALML5, which is closely associated with recurrence and survival outcomes in patients with HPV‐related cancers such as head‐and‐neck carcinoma and oropharyngeal cancer. Thus, this unique CA‐specific epithelial cluster has potential value for prognostic evaluation for CC. (2) Additionally, we identified crucial node genes during disease progression through pseudotime analysis. Of note, we discovered that genes, such as SPRR3 and APOBEC3A, were upregulated during the progression from HPV infection to cervical lesions. According to previous studies, SPRR3 can stimulate cell proliferation,^44^ whereas APOBEC3A can cause virus evolution and cancer mutagenesis by mediating the antiviral response, exerting a ‘second‐hit’‐like effect to accelerate disease escalation.^15^ Moreover, we observed that some progression‐protective genes were downregulated during the transition from HPV infection to cervical lesions. For instance, BPIFB1 was reported to play an important role in the innate immune response^18^, ^19^; hence, BPIFB1 dysfunction potentially facilitated disease progression. Therefore, we propose that early intervention should be considered for HPV infection, including inhibiting second‐hit‐related gene expression and restoring the expression of progression‐protective genes. In terms of the transition from HSIL to CC, the major node genes, such as KRT6C, KRTDAP and S100A7, are extensively related to carcinogenesis, invasion, migration and poor prognosis,^22^, ^23^, ^45^, ^46^ providing novel insights for the accurate diagnosis of CC. Overall, our thorough investigation into the features, functions and differentiation routes of epithelial cell transformation in the normal cervix (both HPV‐positive and HPV‐negative), precancerous lesions and CC provides abundant resources for further scientific research and significant target genes for molecular diagnosis and precise treatment.

In terms of the immune microenvironment of the cervix, we mainly revealed an increase in the exhaustion/cytotoxicity score of CD8+ T cells, imbalance of CD4+ T‐cell subsets, an M1‐ to M2 transition of macrophages and remarkable infiltration and dysfunction of pDCs as the disease escalated from HPV infection, precancerous lesions, and finally to CC. Among them, we found the analysis results regarding pDCs, CD8+ T cells and CD4+ T cells especially noteworthy. (1) Regarding CD8+ T cells, we observed an increased abundance of exhausted T cells within the CC environment, which has been reported in multiple tumours.^47^, ^48^, ^49^ Notably, we discovered that CD8+ Temra cells exerted antiviral effects and cytotoxicity towards tumour cells. According to a previous study, the proportion of CD8+ Temra cells in pancreatic cancer is markedly elevated after treatment with a polyclonal antibody stimulator and PD antibody,^50^ which indicated the significance of CD8+ Temra cells in T‐cell‐mediated tumour death and memory. Thus, investigating how to stimulate and strengthen the antitumour function of CD8+ Temra cells could be a new direction for CC immune therapy research. Additionally, we noticed that CD8+ MAITs were specifically infiltrated within HSIL foci, which have been reported to sense intracellular infection and modulate tissue inflammation.^51^, ^52^, ^53^ Therefore, we propose that CD8+ MAIT cells play a role in immune surveillance. In addition, De Biasi et al.^54^ previously showed that the proportions of MAIT cells were significantly higher in responders with metastatic melanoma to anti‐PD1 therapy, highlighting the potential value of MAITs as biomarkers for patient responses to anti‐PD‐1 therapy. (2) Regarding CD4+ T cells, we discovered a Treg/Th17 imbalance during disease escalation from persistent HPV infection into precancerous lesions and a Treg/Th1‐like imbalance during progression from HSIL to CC. Hence, given the imbalance between CD4+ T‐cell subsets, we propose adoptive T‐cell therapy (tumour reactive CD4+ Th‐cell reinfusion), CAR T‐cell therapy and immune checkpoint blockade, such as anti‐CTLA4, to reactivate Th cells and inhibit Treg cells. (3) Intriguingly, pDCs were reported to play a role in the natural immune response against HPV locally in the cervix,^31^, ^55^ whereas acquired pDC dysregulation could promote tumour progression. Previous studies demonstrated that pDCs could induce the differentiation of naïve CD4+ T cells to Treg cells when cocultured with cervical neoplastic keratinocytes,^56^ indicating the development of a tolerogenic response and interference of an antitumour immune response. Thus, to inhibit the immune tolerance of the CC microenvironment, we could consider pDCs as another promising target in addition to T cells, which have been extensively investigated.

CellphoneDB analysis allowed us to elucidate the cellular crosstalk within the cervical microenvironment, whereas ST analysis further provided the spatial distribution of cellular interactions. Notably, we found several ligand–receptor pairs unique to different disease stages, including CSF3–CSF1R/CSF3R specifically in HPV‐infected normal cervix and JAG1–CD46 predominantly in HSIL, CEACAM5–CD1D remarkably in CC. Among them, CSF3–CSF1R/CSF3R interactions serve as granulocyte colony‐stimulating factors and regulate the innate immune response in the early phase of HPV infection. Regarding the JAG1–CD46 interaction in HSIL, CD46 is a multifunctional protein best known for binding many pathogens to negatively regulate the innate immune system and inducing an immunosuppressive phenotype in T cells,^57^, ^58^ which symbolizes the emergence of immunoregulatory features. In addition, CEACAM5 is overexpressed in various carcinomas, and it is one of the target genes for HPV gene integration,^48^ indicating the role of CEACAM5 as a CC‐associated antigen. Given that CEACAM5–CD1D communication has been reported to facilitate antigen presentation yet activate CD8+ regulatory T cells in the CC TME, we hypothesized that CEACAM5 might serve as a crucial treatment target for HPV‐related CC. In brief, combining cellular interaction analysis with spatial mapping of histological slides allowed us to not only depict a more authentic microenvironment of the cervix during different disease stages but also identify more credible treatment targets for CC.

**FIGURE 8:**
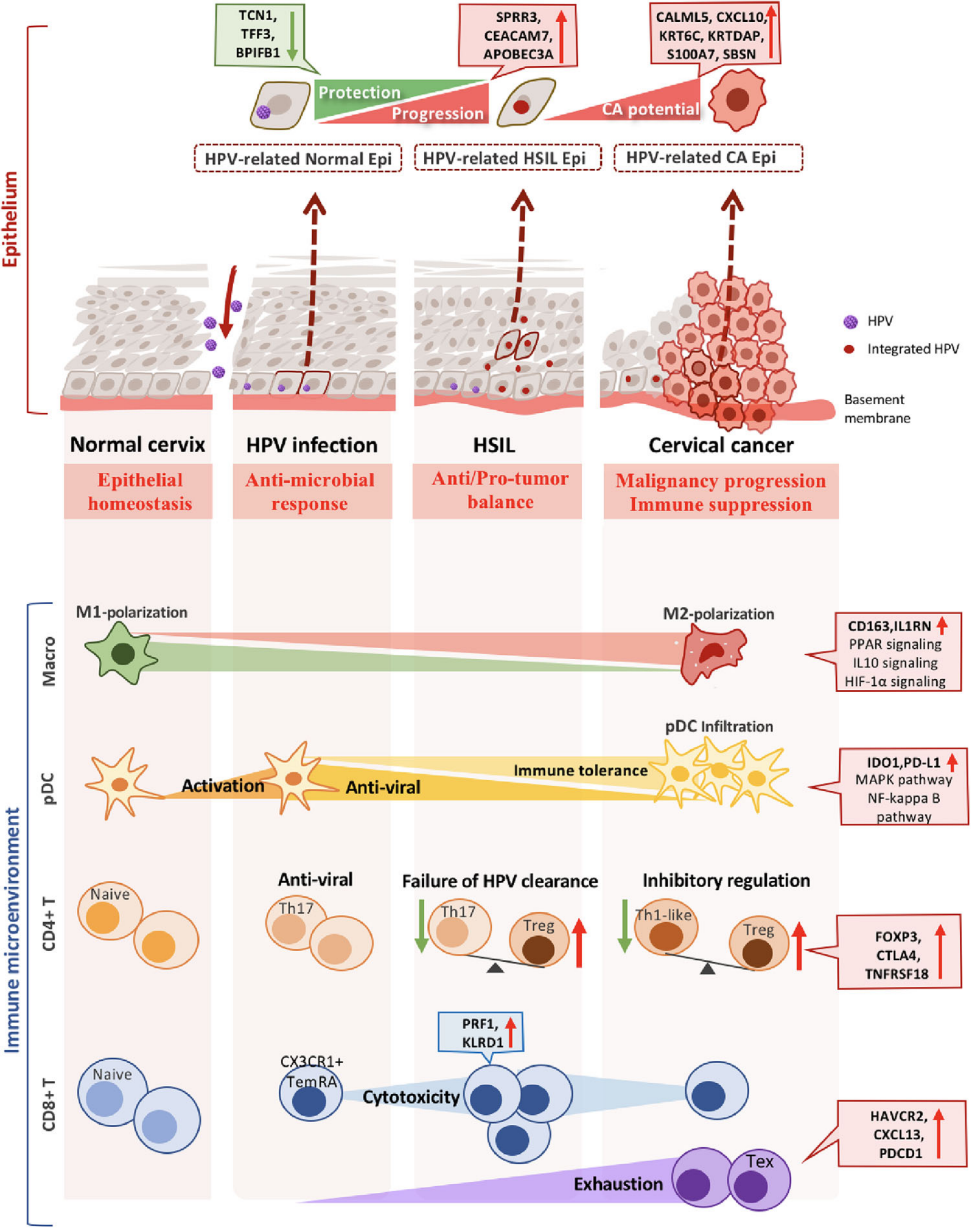
The schematic diagram indicating the mechanism of human papillomavirus (HPV)‐related cervical cancer carcinogenesis in terms of both the malignant transition of epithelial cells and the remodelling of the immune microenvironment. The upper scheme demonstrating three HPV‐related clusters and detailed node gene changes, respectively, during the four key steps of epithelium malignant transition, from normal cervix, HPV‐infected cervix, high‐grade squamous intraepithelial lesions (HSIL) to cervical cancer. As HPV‐infected cervix gradually developed precancer, three genes were upregulated (TCN1, TFF3, BPIFB1), whereas the other three genes were downregulated (SPRR3, CEACAM7, APOBEC3A). Meanwhile, six genes were upregulated as precancer progressed to cancer (CALML5, CXCL10, KRT6C, KRTDAP, S100A7, SBSN). The lower scheme demonstrating the immune microenvironment changes during the disease escalation. As lesion aggravated, macrophages shifted to M2‐polarization with CD163 and IL1RN upregulated. Plasmacytoid DCs (pDCs) activation in the early HPV infection stage might negatively regulate viral replication, whereas as the HPV infection persisted, CA‐derived pDCs exerted immune tolerance and even immune escape. Regarding T cells, the loss of CD4+ Th17/Treg homeostasis might lead to the failure of HPV clearance and disease progression, whereas an imbalanced CD4+ Th1‐like/Treg axis might lead to carcinogenesis. Additionally, the CD8+ T‐cell subtype was altered and exhibited a progressively immunosuppressive trajectory during the malignant transition, the abundance of CD8+ Temra exhibiting a positive immune reaction gradually decreased, and the infiltration of CD8+ Tex cells demonstrating immune dysfunction propelled the malignant program.

In conclusion, we pioneered both the temporal transition and spatial distribution of the cellular subsets during disease progression from normal cervical tissues (including both HPV‐infected and non‐HPV‐infected) and precancerous lesions to CC by integrating scRNA‐seq with ST analysis (Figure 8). Strikingly, we not only innovatively identified three HPV‐related epithelial clusters unique to HPV infection, HSIL and CC but also discovered critical node genes that potentially determined disease progression, further unveiling the unknown mechanism of HPV‐mediated carcinogenesis. In addition, gradual transition of multiple immune cells from a positive immune response to dysregulation and exhaustion led to an immune‐suppressive microenvironment of CC. Finally, the cellular interaction and ST combined analysis further verified a ‘homeostasis‐balance‐malignancy’ change within the cervical microenvironment during disease escalation. However, this study has some potential limitations that could be addressed in future research, the sample size was relatively small, only HPV types 16/18 were included, and lack of validation of in vitro and in vivo experiments. Collectively, these findings not only provide novel insights into HPV‐related cervical carcinogenesis but also unprecedented possibilities for the accurate diagnosis, precise treatment and prognostic evaluation of CC.

## CONFLICT OF INTEREST STATEMENT

The authors declare no conflicts of interest.

## Supporting information

Supporting Information

## Data Availability

Complete scRNA and ST‐sequencing data are available online through the Gene Expression Omnibus (GEO) portal under project accession number GSE208653 and GSE208654. All other data supporting the findings of this study are available from the corresponding authors upon reasonable request.
