# Supplementary material for: Spatiotemporally deciphering the mysterious mechanism of persistent HPV‐induced malignant transition and immune remodelling from HPV‐infected normal cervix, precancer to cervical cancer: Integrating single‐cell RNA‐sequencing and spatial transcriptome
Source: Clin Transl Med. 2023 Mar 26;13(3):e1219. doi: 10.1002/ctm2.1219 (PMC10040725; doi:10.1002/ctm2.1219)
Supplement: Supplementary file 1 — Supporting Information [file CTM2-13-e1219-s001.pdf]

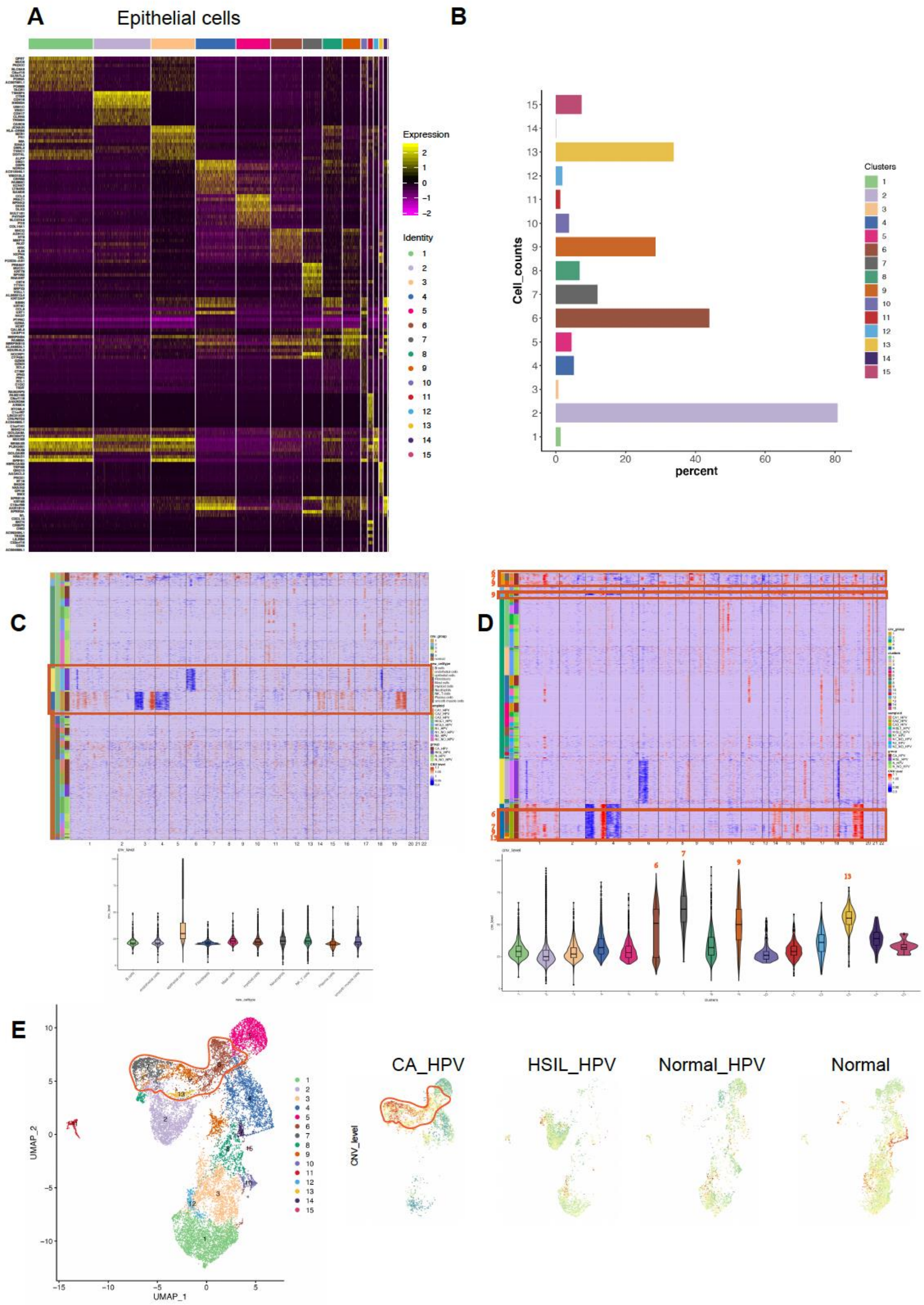

A
Epithelial cells
Expression
Identity
B
Cell_counts
percent
Clusters
C
D
E
UMAP_1
UMAP_2
CA_HPV
HSIL_HPV
Normal_HPV
Normal
CNV_level

Fig S1 The molecular profiles of cervical epithelial cells

(A) The heatmap revealing the top genes of 16 epithelial clusters by unsupervised clustering.

(B) The barplot manifesting the percentage of HPV-infected cells per cluster.

(C)The heatmap and violin plot manifesting the inferred CNV levels of 10 cell types.

(D) The heatmap and violin plot manifesting the inferred CNV levels of 16 epithelial cell clusters.

(E) The UMAP plot manifesting the epithelial cells in 15 clusters (left); The UMAP plot of the epithelial cells indicating CNV levels within the 4 clinical groups respectively including normal cervix without HPV infection, HPV-infected normal cervix, HPV-infected HSIL and HPV-infected cervical cancer (right).

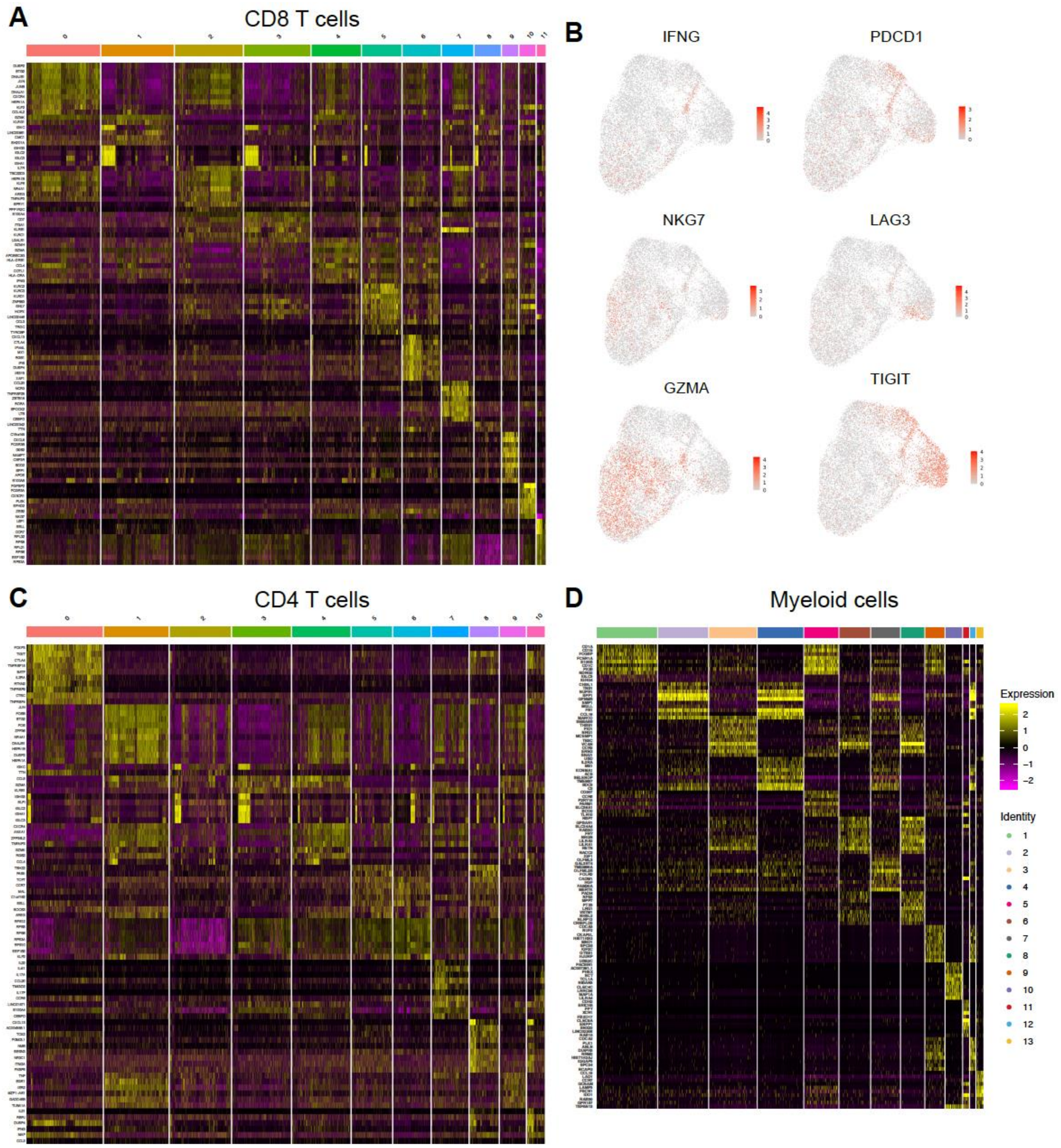

Fig S2 The molecular profiles of immune cells in the microenvironment of cervix

(A) The heatmap revealing the top genes of 12 CD8+ T cell clusters by unsupervised clustering.

(B) The UMAP feature plots of IFNG, NKG7, GZMA representing the cytotoxicity of CD8+ T lymphocytes and PDCD1, LAG3, TIGIT representing the exhaustion of CD8+T lymphocytes.

(C) The heatmap revealing the top genes of 11 CD4+ T cell clusters by unsupervised clustering.

(D) The heatmap revealing the top genes of XX myeloid cell clusters by unsupervised clustering.

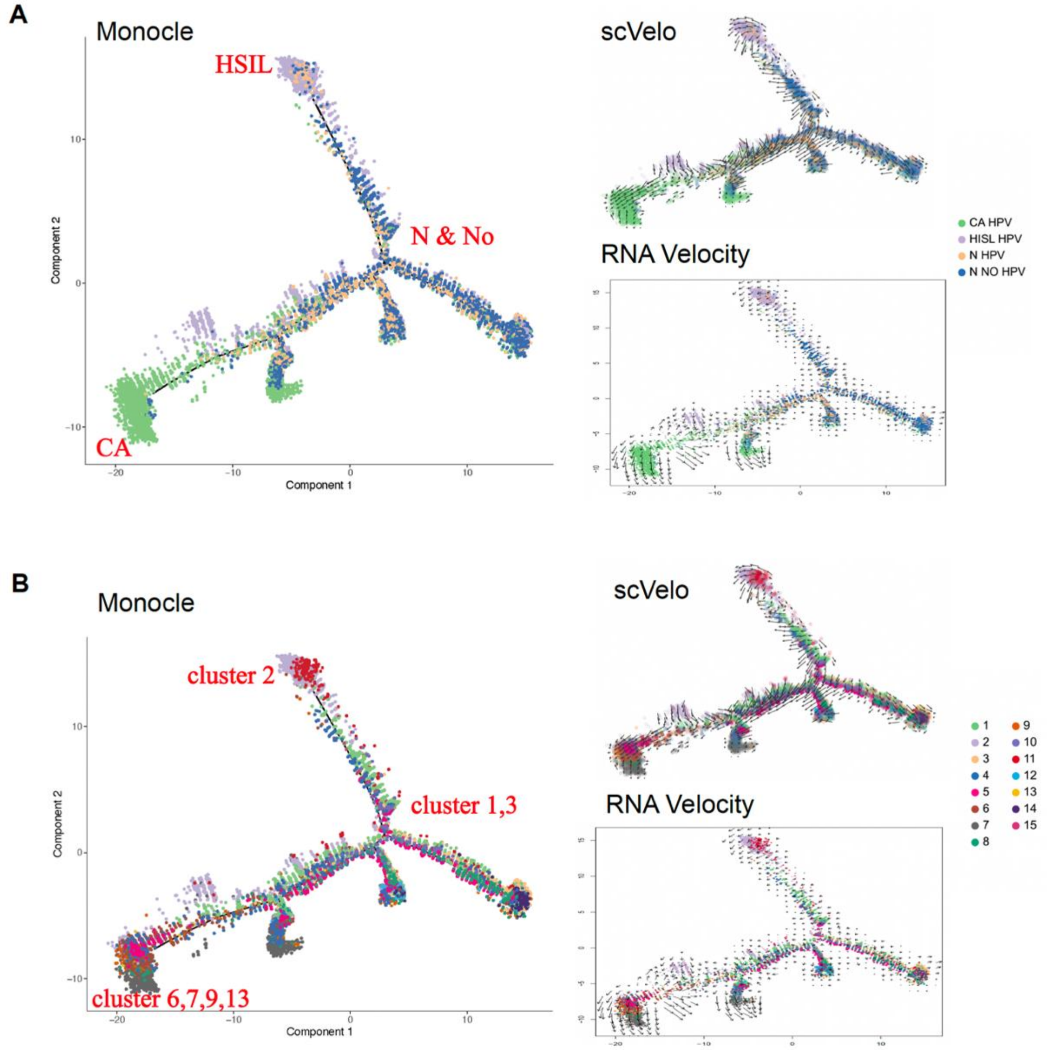

C

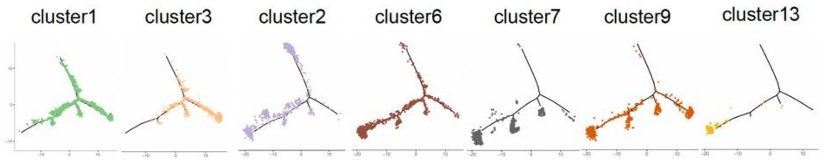

D

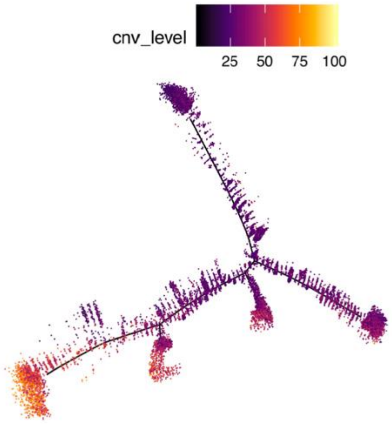

Fig S3 The monocle, scVelo and RNA-velocity of cell clusters in epithelial cells

(A) The pseudotime plots demonstrating the monocle, scVelo and RNA-velocity of of epithelial cells by 4 group

(B) The pseudotime plots demonstrating the monocle, scVelo and RNA-velocity of of epithelial cells by 15 clusters

(C) The pseudotime plots demonstrating the monocle path of cluster 1, 3, 2, 6, 7, 9 and 13

(D) The pseudotime plots demonstrating the trajectory mapped with CNV levels

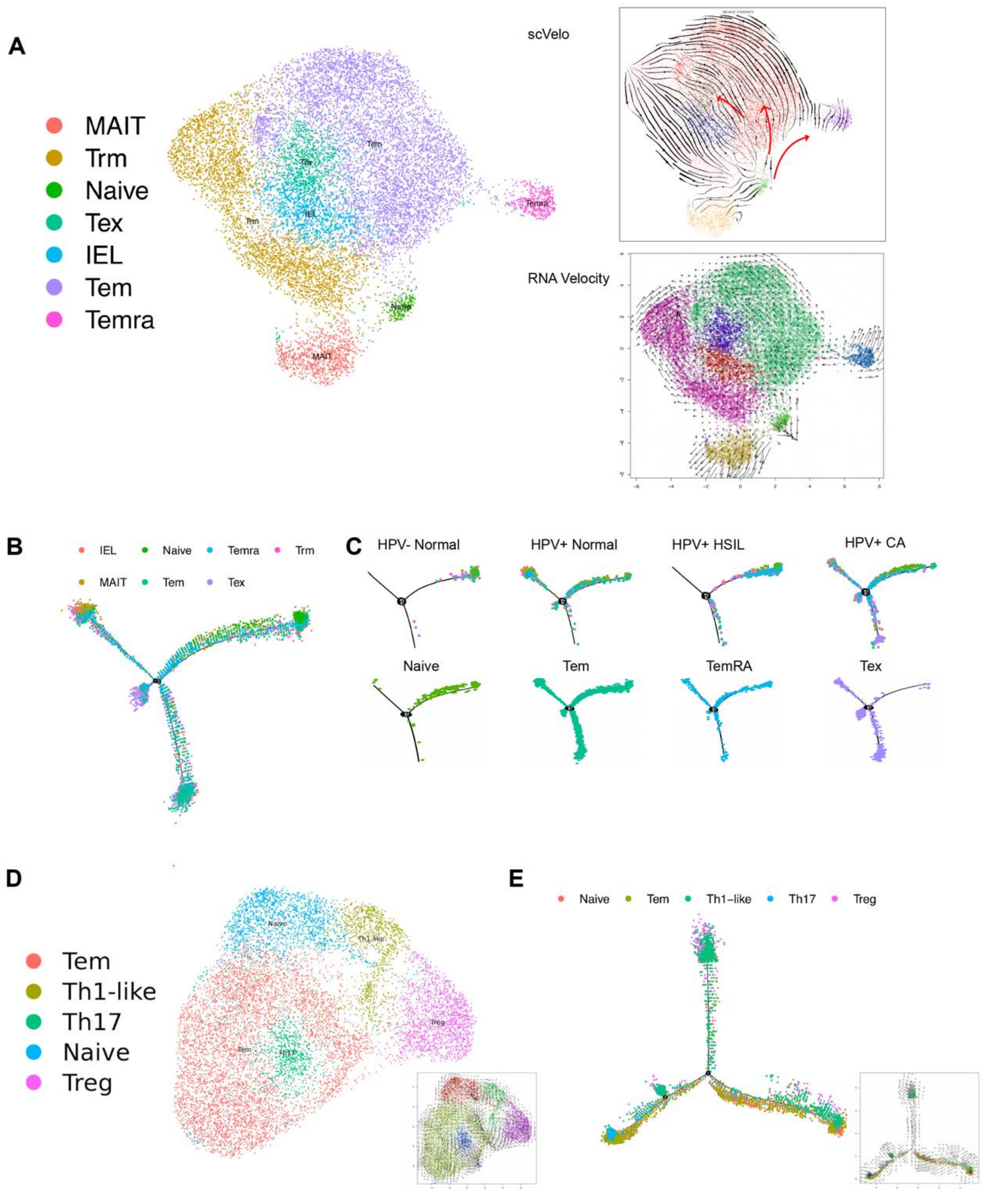

A
MAIT
Trm
Naive
Tex
IEL
Tem
Temra
scVelo
RNA Velocity
B
IEL
Naive
Temra
Trm
MAIT
Tem
Tex
C
HPV- Normal
HPV+ Normal
HPV+ HSIL
HPV+ CA
Naive
Tem
TemRA
Tex
D
Tem
Th1-like
Th17
Naive
Treg
E
Naive
Tem
Th1−like
Th17
Treg

Fig S4 The monocle, scVelo and RNA-velocity of cell subpopulations in CD4 and CD8 cells

(A) The UMAP plots demonstrating the scVelo and RNA-velocity of different CD8 cell types.

(B) The pseudotime plots demonstrating the monocle path of CD8 cells by group

(C) The pseudotime plots demonstrating the monocle path of CD8 cells by cell subtypes.

(D) The UMAP plots demonstrating the RNA-velocity of different CD4 cell types.

(E) The pseudotime plots demonstrating the RNA-velocity of different CD4 cell types.

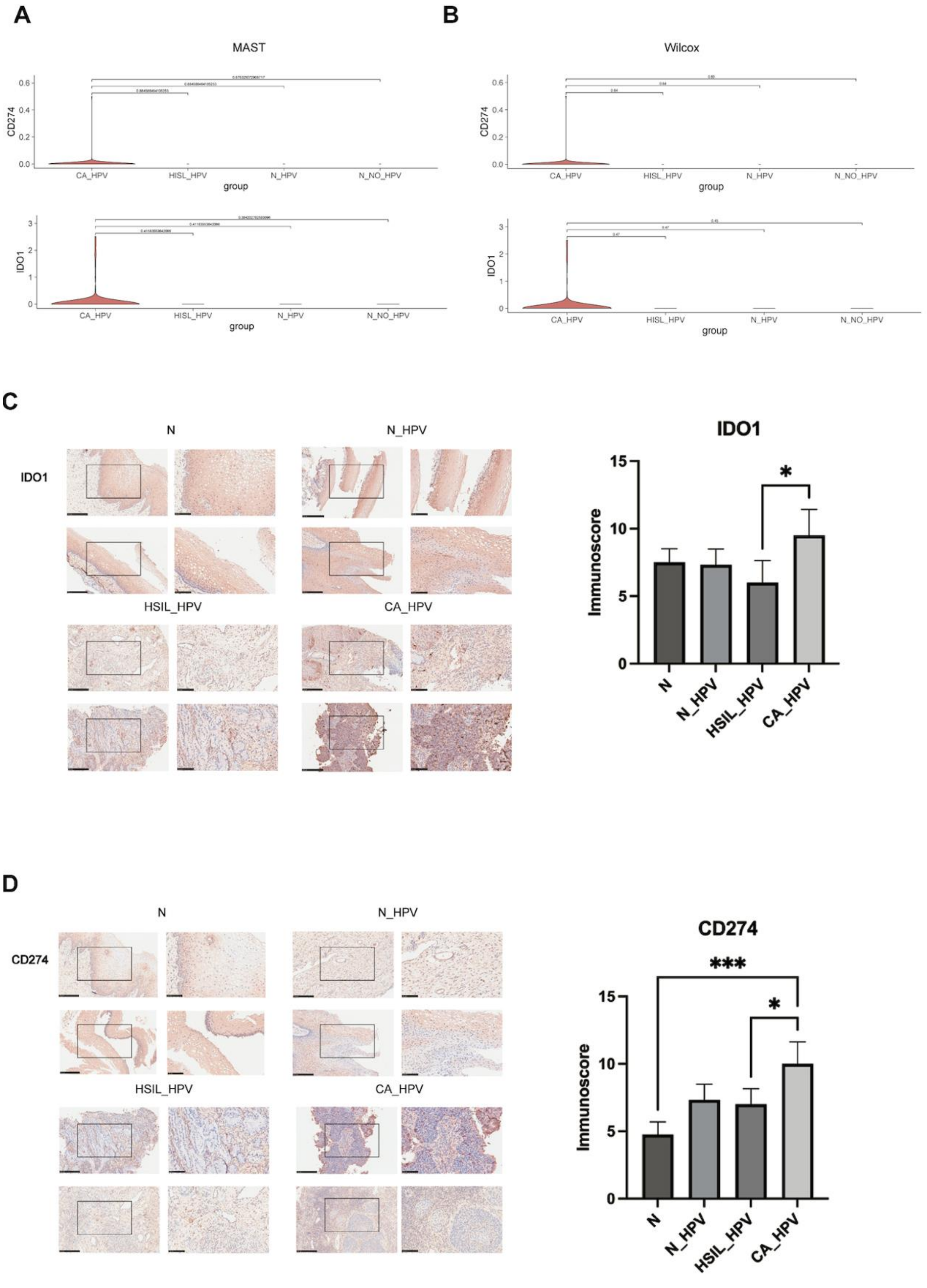
A
B
MAST
Wilcox
CD274
IDO1
group
CA_HPV
HISL_HPV
N_HPV
N_NO_HPV
C
D
N
N_HPV
HSIL_HPV
CA_HPV
IDO1
CD274
Immunoscore
*
***

Fig S5 The expression of CD274 and IDO1 in pDCs among four clinical groups.

(A) The violin plots manifesting the expression of CD274 (upper) and IDO1 (lower) in pDCs of the 4 clinical groups calculated by MAST algorithm.

(B) The violin plots manifesting the expression of CD274 (upper) and IDO1 (lower) in pDCs of the 4 clinical groups calculated by wilcox algorithm.

(C) Representative images of IDO1 IHC staining in human cervical specimens among four clinical groups. Scale bars, 50 μm; The immuno-score comparison among four clinical groups by ANOVA test.

(D) Representative images of CD274 IHC staining in human cervical specimens among four clinical groups. Scale bars, 50 μm. The immuno-score comparison among four clinical groups by ANOVA test.

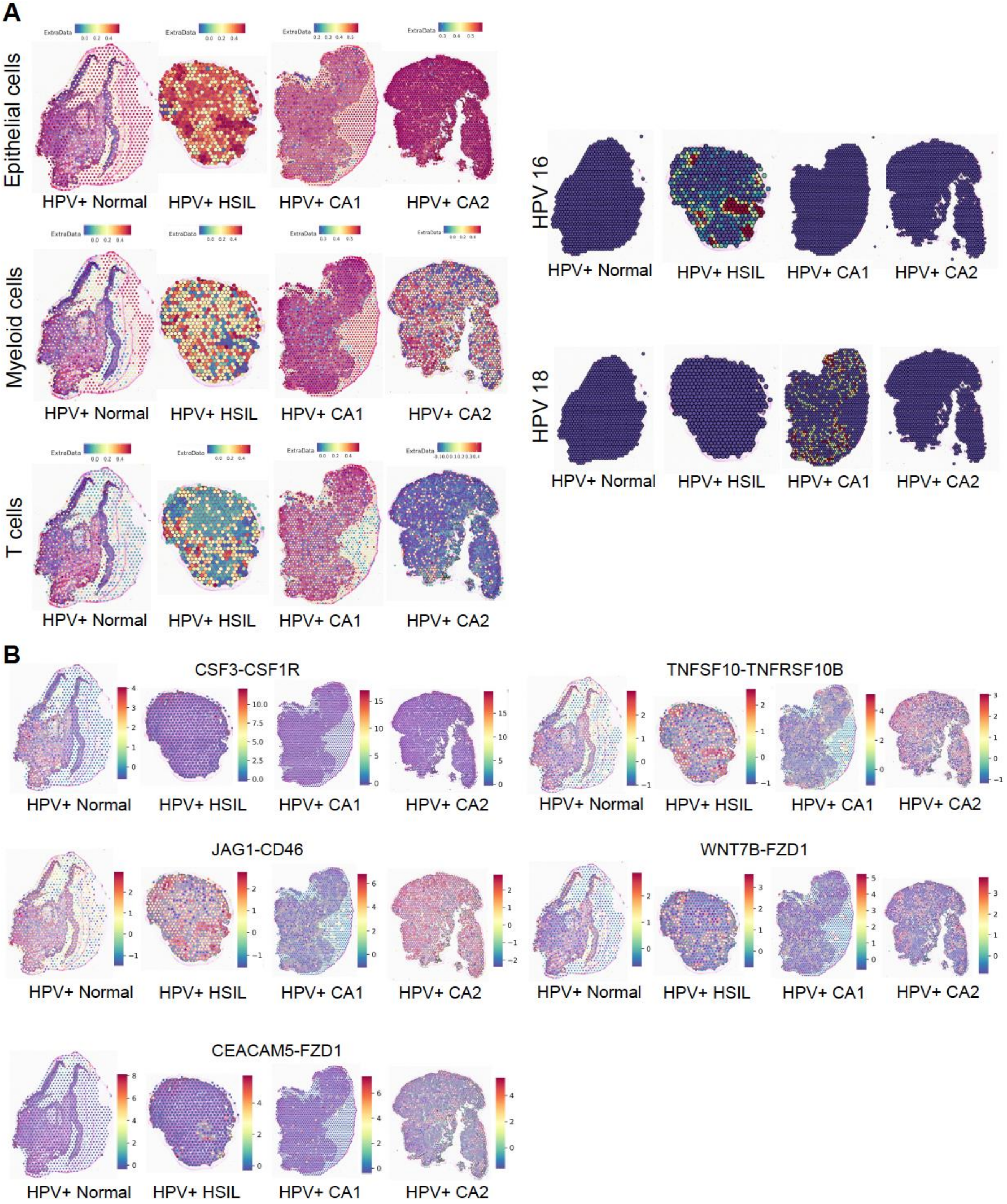

A
Epithelial cells
Myeloid cells
T cells
HPV+ Normal
HPV+ HSIL
HPV+ CA1
HPV+ CA2
HPV 16
HPV 18
B
CSF3-CSF1R
TNFSF10-TNFRSF10B
JAG1-CD46
WNT7B-FZD1
CEACAM5-FZD1

Fig S6 The spatial profiles of specific cell subpopulations and cellular crosstalk in the microenvironment of cervix

(A) The spatial plots demonstrating the projection of epithelial cells (upper left), myeloid cells (medium left) and T cells (lower left) as well as HPV16-infected cells (upper right) and HPV 18-infected cells (lower right) based on scRNA-seq results within the HPV-positive normal cervix (SYN), HPV-positive HSIL (NYY) and 2 HPV-positive cervical cancer tissues (SYM, LYH).

(B) The spatial plots demonstrating the projection of cellular interaction including CSF3-CSF1R, JAG1-CD46, CEACAM5-FZD1, TNFSF10-TNFRSF10B and WNT7B-FZD1 based on scRNA-seq results within the HPV-positive normal cervix (SYN), HPV-positive HSIL (NYY) and 2 HPV-positive cervical

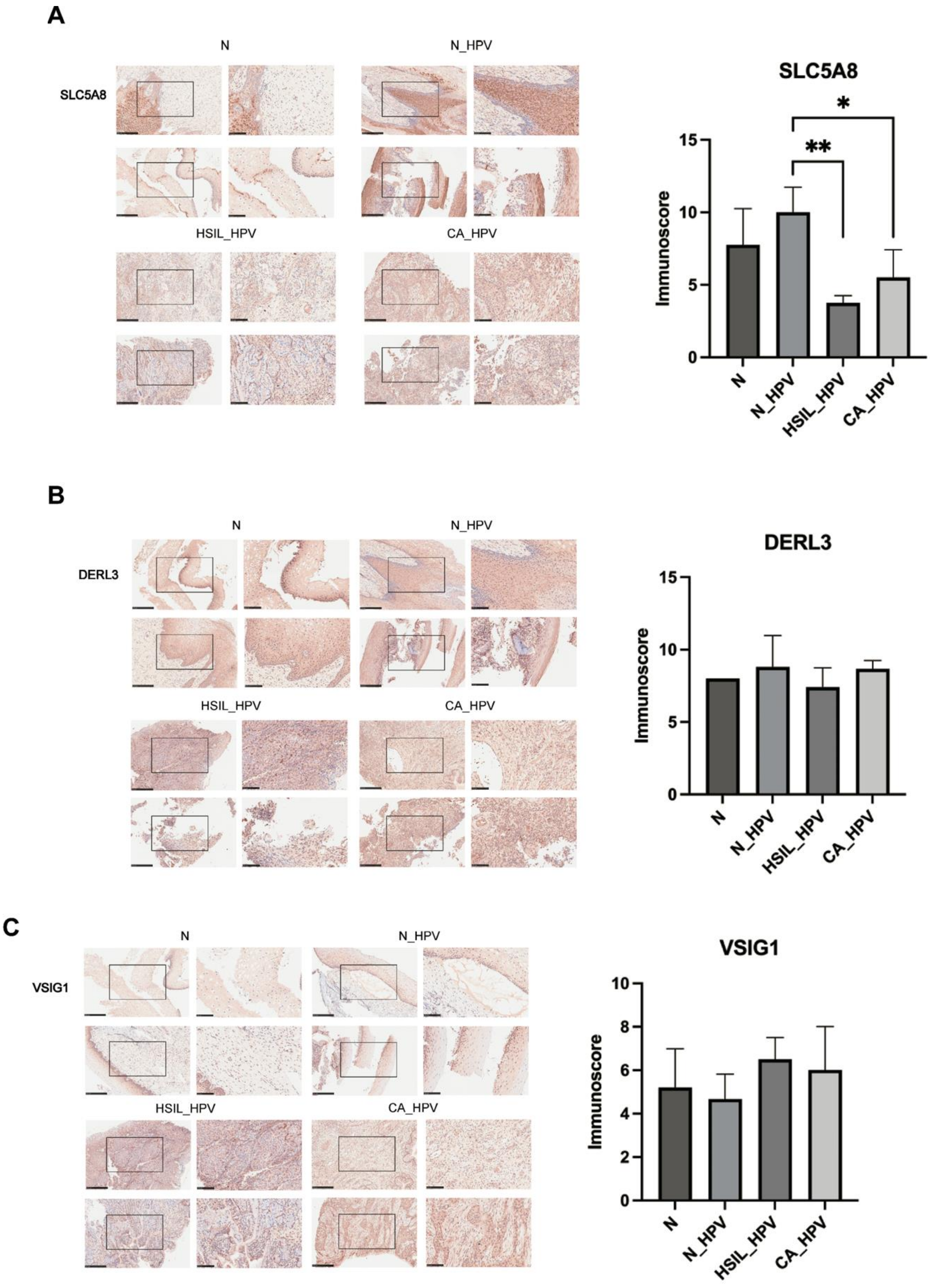
A
N
N_HPV
SLC5A8
HSIL_HPV
CA_HPV
SLC5A8
*
**
Immunoscore
15
10
5
0
N
N_HPV
HSIL_HPV
CA_HPV
B
N
N_HPV
DERL3
HSIL_HPV
CA_HPV
DERL3
Immunoscore
15
10
5
0
N
N_HPV
HSIL_HPV
CA_HPV
C
N
N_HPV
VSIG1
HSIL_HPV
CA_HPV
VSIG1
Immunoscore
10
8
6
4
2
0
N
N_HPV
HSIL_HPV
CA_HPV

D

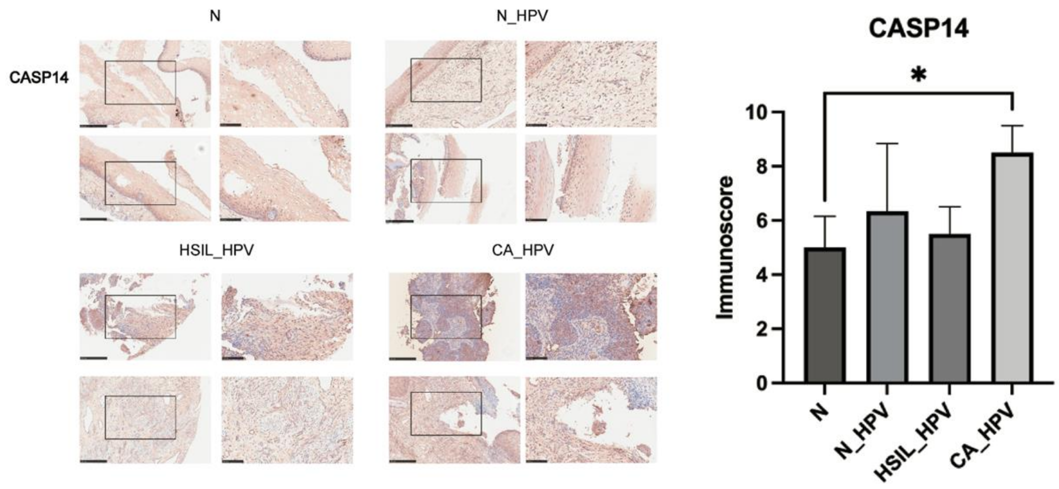

E

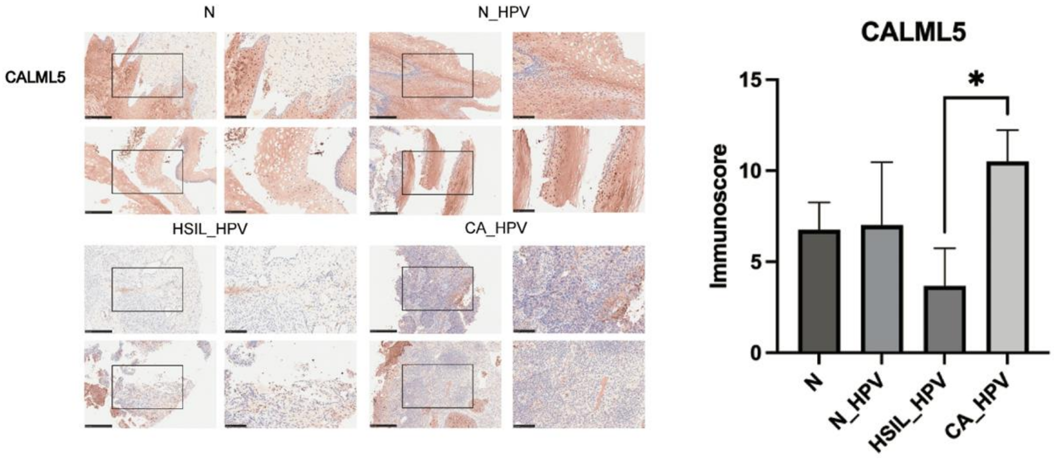

Fig S7 The expression of markers in three HPV-related clusters among four clinical groups.

(A) Representative images of SLC5A8 IHC staining in human cervical specimens among four clinical groups. Scale bars, 50 μm; Immuno-score comparison among four clinical groups by ANOVA test.

(B) Representative images of DERL3 IHC staining in human cervical specimens among four clinical groups. Scale bars, 50 μm; Immuno-score comparison among four clinical groups by ANOVA test.

(C) Representative images of VSIG1 IHC staining in human cervical specimens among four clinical groups. Scale bars, 50 μm; Immuno-score comparison among four clinical groups by ANOVA test.

(D) Representative images of CASP14 IHC staining in human cervical specimens among four clinical groups. Scale bars, 50 μm; Immuno-score comparison among four clinical groups by ANOVA test.

(E) Representative images of CALML5 IHC staining in human cervical specimens among four clinical groups. Scale bars, 50 μm; Immuno-score comparison among four clinical groups by ANOVA test.

Methods

Patient and tissue sample collection

This study complied with all the relevant ethical regulations, and the protocols were approved by the institutional review boards of Fudan University Obstetrics and Gynecology Hospital. The study involved 9 patients, including 2 healthy patients, 2 patients diagnosed with HPV infection and normal histology, 2 patients diagnosed with HSIL and 3 patients diagnosed with cervical cancer. All patients were aged 35-65 years old, did not have other comorbidities or receive any treatment before, had not been vaccinated against HPV, and had no family history of any malignant disease. Persistent HPV infection was limited to type 16 or 18. Those with HPV infection less than one year were excluded. Cervical samples were obtained from each patient

during colposcopy examination, and the diagnose were confirmed by the histopathological examination by expert pathologists. After resection, the samples were collected and transferred for tissue preparation.

Preparation of single-cell suspensions

Samples from normal, HPV-infected, HSIL and cervical cancer tissues from nine resection specimens were isolated and transported rapidly to the research facility. Each sample was subsequently minced on ice to pieces less than 1 cubic millimeters in size, followed by enzymatic digestion with manual shaking every 5 min. The samples were then centrifuged at 300 rcf for 30 sec at room temperature, and the supernatants were removed without disturbing the cell pellets. Next, 1× PBS (calcium- and magnesium-free) supplemented with 0.04% weight/volume BSA (400 μg/ml) was added, and then, the samples were centrifuged at 300 rcf for 5 min. The cell pellets were resuspended in 1 ml red blood cell lysis buffer and incubated for 10 min at 4°C. After red blood cell lysis, the samples were resuspended in 1 ml PBS supplemented with 0.04% BSA. Next, the samples were filtered with Scienceware Flowmi 40-μm cell strainers (VWR). After tumor dissociation, cell concentration and cell viability were determined with a hemocytometer and Trypan Blue staining.

Preparation of ST samples

Tissue preparation, cryosectioning, fixation, staining and brightfield imaging for ST

The tissues obtained from the cervix were cut into 4-5 mm^3 pieces and treated with cleanroom wipes. After removing blood from the tissue surface, fresh tissue was embedded with OCT and snap frozen at -80°C. Cryosections were cut at 10-μm thickness and mounted onto ST arrays. Next, the tissues were dehydrated with isopropanol for 1 min followed by staining with H&E. Slides were mounted in 80% glycerol, and brightfield images were captured on a 3D HISTECH Pannoramic MIDI FL whole-slide scanner at 40× resolution.

ST barcoded microarray slide information

Library preparation slides used were purchased from the Spatial Transcriptomics team (https://www.10xgenomics.com/). Each of the spots printed onto the array was 55 μm in diameter and 100 μm from center to center, covering an area of 6.5×6.5 mm2. Each slide included 4 capture areas, each with ~5,000 unique gene expression spots.

Tissue optimization

Tissue sections were placed on the corresponding Capture Areas on the Visium Spatial Tissue Optimization Slide. The sections were fixed, stained, and permeabilized for different lengths of time. mRNA molecules that were released during permeabilization bound to capture probes on the slide. cDNA was generated using fluorescently labeled nucleotides to visualize the synthesized cDNA. Finally, the tissue was enzymatically removed, leaving behind the fluorescently labeled cDNA that could be visualized using fluorescence microscopy to select the optimal permeabilization time. i. The tissue was cryosectioned and placed on the Capture Areas

of a Visium Spatial Tissue Optimization slide. The tissue sections were then fixed with methanol and stained with hematoxylin and eosin (H&E). Hematoxylin stains the nuclei of mammalian cells, while eosin stains the extracellular matrix and cytoplasm. The stained tissue sections were imaged, and the final fluorescent images were compared[60]. ii. The tissue sections were permeabilized for varying amounts of time. Each Capture Area bound polyadenylated mRNA from the attached tissue section. A Master Mix containing reverse transcription (RT) reagents and fluorescently labeled nucleotides was added on top of the tissue sections, resulting in fluorescently labeled cDNA. iii. The tissue was enzymatically removed, leaving behind the fluorescent cDNA covalently linked to oligonucleotides on the Visium Spatial Tissue Optimization slide. Fluorescent cDNA was visualized under fluorescence imaging conditions that were determined using the Visium Imaging Test Slide. H&E and fluorescence images are compared. The permeabilization time that resulted in the maximum fluorescence signal with the lowest signal diffusion was considered the optimal time point. If the signal was the same at two time points, the longer permeabilization time was considered to be the optimal time.

On-slide tissue permeabilization, cDNA synthesis, library construction and sequencing

the tissue sections that were placed on these Capture Areas were fixed, stained, and permeabilized, and cellular mRNA was captured by the primers on the gene expression spots. All the cDNA molecules that were generated from the mRNA captured by the primers on a specific spot shared a common Spatial Barcode. Libraries were generated from the cDNA and sequenced, and Spatial Barcodes are used to associate the reads back to the images of specific tissue sections

for spatial gene expression mapping. i. A Permeabilization Enzyme was used to permeabilize the fixed and stained tissue sections on the slides. The polyadenylated mRNA released from the overlying cells was captured by the primers on the spots. RT Master Mix containing reverse transcription reagents was added to the permeabilized tissue sections. Incubation with the reagents resulted in the production of spatially barcoded, full-length cDNA from polyadenylated mRNA on the slide. ii. Second Strand Mix was added to the tissue sections on the slide to initiate second strand synthesis. This step was followed by the denaturation and transfer of the cDNA from each Capture Area to a corresponding tube for amplification and library construction. iii. After the transfer of cDNA from the slide, spatially barcoded, full-length cDNA was amplified via PCR to generate sufficient mass for library construction. ⅳ. Enzymatic fragmentation and size selection were used to optimize the cDNA amplicon size. P5, P7, i7 and i5 sample indexes and TruSeq Read 2 (read 2 primer sequence) were added via End Repair, A-tailing, Adaptor Ligation, and PCR. The final libraries contained the P5 and P7 primers used in Illumina amplification.

Visium Spatial Gene Expression libraries comprise standard Illumina paired-end constructs that are flanked with P5/P7, which are necessary for binding to the Illumina flow cell. TruSeq Read 1 was used for priming and sequencing the 16 bp Spatial Barcode and 12 bp UMI, and TruSeq Read 2 was used for priming and sequencing the cDNA insert. The two 10 bp sample indexes are sequenced in the i5 and i7 reads. Calculating sequencing depth requires estimating the approximate Capture Area (%) that was covered by tissue. This may be performed visually or by using the Visium Manual Alignment Wizard in Loupe Browser to obtain more accurate

measurements. See the examples below in which the coverage area was visually estimated. A minimum of 50,000 read pairs per tissue-covered spot were estimated on the Capture Area.

Single-cell RNA-seq data preprocessing

The Cell Ranger software pipeline (version 3.1.0) provided by 10× Genomics was used to demultiplex cellular barcodes, map reads to the genome and transcriptome using the STAR aligner, and downsample reads as required to generate normalized aggregate data across samples, producing a matrix of gene counts versus cells. We processed the unique molecular identifier (UMI) count matrix using the R package Seurat (version 3.1.1)[61]. To remove low-quality cells and likely multiple captures, which is a major concern in microdroplet-based experiments, we applied criteria to remove cells with UMI/gene numbers out of the limit of mean value +/- 2-fold of standard deviations assuming a Gaussian distribution of the UMI/gene numbers of each cell. After the visual inspection of the distribution of cells based on the fraction of mitochondrial genes expressed, we further discarded low-quality cells where >10% of the counts belonged to mitochondrial genes. After applying these QC criteria, a total of 5510~15372 single cells remained and were included in downstream analyses. There are 1334~3142 genes per cell, and 3547~12940 UMI per cell. Library size normalization was performed in Seurat on the filtered matrix to obtain the normalized count.

The top genes whose expression was most variable across single cells were identified using the method described in Macosko et al.[62] Briefly, the average expression and dispersion were calculated for each gene, and genes were subsequently placed into ten bins based on

expression. Principal component analysis (PCA) was performed to reduce the dimensionality of the log-transformed gene-barcode matrices of the top variable genes. The cells were clustered based on a graph-based clustering approach and visualized in 2 dimensions using UMAP. A likelihood ratio test that simultaneously tested for changes in the mean expression and in the percentage of cells expressing a gene was used to identify significantly differentially expressed genes between clusters. Here, we use the R package SingleR, which is a novel computational method for unbiased cell type recognition of scRNA-seq, with the reference transcriptomic datasets 'Human Primary Cell Atlas'[63] to infer the origin of each of the single cells independently and to identify cell types.

Differentially expressed genes (DEGs) were identified using the FindMarkers function of Seurat package. A P value < 0.05 and |log2foldchange| > 0.58 were set as the thresholds that defined significantly differential expression. GO enrichment and KEGG pathway enrichment analyses of the DEGs were performed using R based on the hypergeometric distribution. In addition, ggplot2 package was used to draw violin diagram of the expression levels of different genes in pDC, MAST and wilcox package was used to calculate P values.

ST 10X Visium data preprocessing

Raw FASTQ files and histology images were processed for each sample with SpaceRanger software version 1.2.0, which uses STAR for genome alignment, and the samples were aligned with the hg38 reference genome. We processed the unique molecular identifier (UMI) count matrix using the R package Seurat and normalized the data by SCTransform to account for

variance in sequencing depth across data points. The variance in molecular counts per spot can be substantial in spatial datasets, particularly if there are differences in cell density across the tissue. This variance in molecular counts across spots is not just technical in nature, but it is also dependent on tissue anatomy. Therefore, we included all the spots in our analysis.

The top genes whose expression was most variable across single cells were identified using the method described by Macosko et al.[62] Briefly, the average expression and dispersion were calculated for each gene, and genes were subsequently placed into ten bins based on expression. Principal component analysis (PCA) was performed to reduce the dimensionality of the log-transformed gene-barcode matrices of the most variable genes. Cells were clustered based on a graph-based clustering approach and visualized in 2 dimensions using UMAP.

To perform differential expression (DGE) based on preannotated anatomical regions within the tissue, the FindMarkers function of the Seurat[61] package was applied, and the default method was 'wilcox'. A P value < 0.05 and $|\log2foldchange| > 0.58$ were set as the thresholds to identify significantly differential expression. GO enrichment and KEGG pathway enrichment analyses of the DEGs were performed using R based on the hypergeometric distribution.

We used SPOTlight[64] to integrate 10X Visium with scRNA-seq data to determine the location of cell types and states within a complex tissue.

Immunohistochemistry (IHC) and the Immunocore

Cervical tissues from human models were collected and fixed in 10% formalin overnight and embedded in paraffin. IDO1 Polyclonal antibody, PD-L1/CD274 Monoclonal antibody,

SLC5A8 Polyclonal antibody, Rabbit Anti-DERL3 antibody, VSIG1 Polyclonal antibody, Rabbit Anti-Caspase-14 p10 antibody and CALML5 Polyclonal antibody were used for IHC staining. IHC staining were scored separately by two independent pathologists. The IHC staining results were assigned a mean score considering both the intensity of staining and the proportion of tumor cells with an unequivocal positive reaction. Each section was independently assessed by 2 pathologists without prior knowledge of patient data. Positive reactions were defined as those showing brown signals in the cell cytoplasm, a staining index (value 0-12) was determined by multiplying the score for staining intensity with the score for positive area. The intensity was scored as follows: 0, negative;1, weak;2, moderate; and 3, strong. The frequency of positive cells was defined as follows: 0, less than 5%; 1, 5% to 25%; 2, 26% to 50%; 3, 51%to 75%; and 4, greater than 75%. When the staining was heterogeneous, we scored it as follows: each component was scored independently and summed for the results. For example, a specimen containing 75% tumor cells with moderate intensity (3*2=6) and another 25% tumor cells with weak intensity (1*1=1) received a final score of 6+1=7. A-NOVA test was used for immune-score comparison among four groups.

Advanced analysis method

Cell cycle analysis

Prediction of the cell cycle phase of individual cells was performed in Seurat[61] with the Cell Cycle Scoring function. Briefly, genetic markers associated with G2/M and S phase were used to

assign cell scores, and cells expressing neither of the G2/M nor S phase markers were classified as being in G1 phase.

Pseudotime analysis

We determined the developmental pseudotime with the Monocle2 (v2.9.0) package[65]. The raw count was first converted from the Seurat object into the CellDataSet object with the import CDS function in Monocle. The estimateSizeFactors and estimateDispersions function in Monocle2 package were used to estimate size factors and dispersions of the data. Then the detectGenes function in Monocle2 package was performed to filter low-quality cells. The genes expressed in at least 10 cells of the dataset were kept. We used the differentialGeneTest function in Monocle2 package to select ordered genes (qval < 0.01) that were likely to be informative in the ordering of cells along the pseudotime trajectory. The setOrderingFilter function marks genes that will be used for clustering in subsequent calls to clusterCells. The dimensional reduction clustering analysis was performed with the reduceDimension function(max_components = 2,verbose = T,check_duplicates = F), followed by trajectory inference with the orderCells function using default parameters. Gene expression was plotted with the plot_genes_in_pseudotime function to track changes over pseudotime.

SCENIC analysis

The SCENIC analysis was run using the motifs database for RcisTarget and GRNboost (SCENIC[66] version 1.1.2.2, which corresponds to RcisTarget 1.2.1 and AUCell 1.4.1) with the

default parameters. In detail, the runSCENIC_1_coexNetwork2modules function was first performed to convert the output from GENIE3/GRNBoost to co-expression modules. Then, the runSCENIC_2_createRegulons(coexMethod = top10perTarget) was used to prune co-expression modules using TF-motif enrichment analysis. The runSCENIC_3_scoreCells function was finally used to score the regulons on the individual cells, we identified transcription factor (TF)-binding motifs that were overrepresented on a gene list with the RcisTarget package. The activity of each group of regulons in each cell was scored by the AUCell package.

To evaluate the cell type specificity of each predicted regulon, we calculated the regulon specificity score (RSS) with the calculate_rrs function in scFunctions(https://github.com/FloWuenne/scFunctions/) package for all regulons over all cell types. The RSS was visualized by performing the plot_rrs_ranking function on the RSS scores with the most specific regulons ranking the highest per cell type; this score was based on the Jensen–Shannon divergence (JSD), which is a measure of the similarity between two probability distributions. Specifically, we calculated the Jensen–ShannonJensen-Shannon divergence (JSD) between each vector of overlapping binary regulon activity with the assignment of cells to a specific cell type[67]. The CSI (connection specificity index) scores were calculated for all regulon pairs based on the AUCs matrix for all regulons with the calculate_csi function in scFunctions package. The regulon modules were visualized using the plot_csi_modules function in scFunctions package using default parameters, (https://github.com/FloWuenne/scFunctions/) package.

Cell–cell communication analysis

We used CellPhoneDB[68] (v2.0) to identify biologically relevant ligand–receptor interactions based on the single-cell transcriptomics (scRNAseq) data. The command which used to perform cell–cell communication analysis was as follows : cellphonedb method statistical-analysis metadata.tsv counts.tsv --counts-data gene_name --iterations=1000 --threshold=0.1 --threads=10 --pvalue=0.05 --output-path=./ --database cellphone.db –verbose.

In detail, we defined a ligand or a receptor as “expressed” in a particular cell type if 10% of the cells of that type had nonzero read counts for the gene encoding a ligand/receptor. Statistical significance was then assessed by randomly shuffling the cluster labels of all cells and repeating the above steps, which generated a null distribution for each LR pair in each pairwise comparison between two cell types. After running 1,000 permutations, P values were calculated with the normal distribution curve generated from the permuted LR pair interaction scores. To define networks of cell–cell communication, we linked any two cell types where the ligand was expressed in the first cell type and the receptor in the second. The R packages Igraph and Circlize were used to display the cell–cell communication networks.

Gene set variation analysis (GSVA)

To perform the Gene Set Variation Analysis, the GSEABase package (version 1.44.0) was used to load the gene set file, which was downloaded and processed from the KEGG database (https://www.kegg.jp/). To assign pathway activity estimates to individual cells, we applied GSVA[69] using standard settings, as implemented in the GSVA package (version 1.30.0). The

differences in pathway activities scored per cell were calculated with the LIMMA package (version 3.38.3).

CNV analysis

Initial CNVs for each region were estimated by the inferCNV[70] R package. The command which used to perform CNV analysis was as follows : run(infercnv_obj, cutoff= 0.1, analysis_mode= 'samples', tumor_subcluster_pval=0.05, hclust_method = 'ward.D2', out_dir='./', num_threads=10, cluster_by_groups=TRUE, denoise=TRUE, no_plot = T, no_prelim_plot = F, HMM=F). In detail, the CNV of total cell types was calculated by the expression level observed in single-cell sequencing data for each cell with a cutoff of 0.1. The genes were sorted based on their chromosomal location, and a moving average of gene expression was calculated using a window size of 101 genes. The expression was then centered to zero by subtracting the mean. The epithelial cells were selected as malignant cells, leaving all the remaining cells as normal cells. Denoising was carried out to generate the final CNV profile.

Methods-only references

60. Moncada, R. et al. Integrating microarray-based spatial transcriptomics and single-cell RNA-seq reveals tissue architecture in pancreatic ductal adenocarcinomas. *Nat. Biotechnol.* 38, 333–342 (2020).

61. Butler, A., Hoffman, P., Smibert, P., Papalexi, E. & Satija, R. Integrating single-cell transcriptomic data across different conditions, technologies, and species. *Nat. Biotechnol.* 36, 411–420 (2018).

62. Macosko, E. Z. et al. Highly parallel genome-wide expression profiling of individual cells using nanoliter droplets. *Cell* 161, 1202–1214 (2015).

63. Aran, D. et al. Reference-based analysis of lung single-cell sequencing reveals a transitional profibrotic macrophage. *Nat. Immunol.* 20, 163–172 (2019).

64. Elosua-Bayes, M., Nieto, P., Mereu, E., Gut, I. & Heyn, H. SPOTlight: seeded NMF regression to deconvolute spatial transcriptomics spots with single-cell transcriptomes. *Nucleic Acids Res.* 49, e50 (2021).

65. Trapnell, C. et al. The dynamics and regulators of cell fate decisions are revealed by pseudotemporal ordering of single cells. *Nat. Biotechnol.* 32, 381–386 (2014).

66. Aibar, S. et al. SCENIC: single-cell regulatory network inference and clustering. *Nat. Methods* 14, 1083–1086 (2017).

67. Suo, S. et al. Revealing the critical regulators of cell identity in the mouse cell Atlas. *Cell Rep.* 25, 1436–1445.e3 (2018).

68. Efremova, M., Vento-Tormo, M., Teichmann, S. A. & Vento-Tormo, R. CellPhoneDB:

inferring cell-cell communication from combined expression of multi-subunit ligand-receptor complexes. *Nat. Protoc.* 15, 1484–1506 (2020).

69. Hanzelmann, S., Castelo, R. & Guinney, J. GSVA: gene set variation analysis for microarray and RNA-seq data. *BMC Bioinform.* 14, 7 (2013).

70. Puram, S. V. et al. Single-cell transcriptomic analysis of primary and metastatic tumor ecosystems in head and neck cancer. *Cell* 171, 1611–1624.e24 (2017).
